# A Miniaturized Screening Platform to Identify Novel Regulators of Extracellular Matrix Alignment

**DOI:** 10.1158/2767-9764.CRC-22-0157

**Published:** 2022-11-22

**Authors:** Caitlin E. Jones, Joe T. Sharick, Steven T. Sizemore, Edna Cukierman, Anne Marie Strohecker, Jennifer L. Leight

**Affiliations:** 1 Department of Biomedical Engineering, The Ohio State University, Columbus, Ohio.; 2 The James Comprehensive Cancer Center, Program in Cancer Biology, The Ohio State University, Columbus, Ohio.; 3 Department of Radiation Oncology, The Ohio State University, Columbus, Ohio.; 4 Cancer Signaling and Epigenetics, The Marvin and Concetta Greenberg Pancreatic Cancer Institute, Fox Chase Cancer Center, Temple Health, Philadelphia, Pennsylvania.; 5 Department of Cancer Biology and Genetics, The Ohio State University, Columbus, Ohio.

## Abstract

Extracellular matrix (ECM) alignment contributes to metastasis in a number of cancers and is a known prognostic stromal factor; however, the mechanisms controlling matrix organization remain unclear. Cancer-associated fibroblasts (CAF) play a critical role in this process, particularly via matrix production and modulation of key signaling pathways controlling cell adhesion and contractility. Stroma normalization, as opposed to elimination, is a highly sought strategy, and screening for drugs that effectively alter ECM alignment is a practical way to identify novel CAF-normalizing targets that modulate ECM organization. To meet this need, we developed a novel high-throughput screening platform in which fibroblast-derived matrices were produced in 384-well plates, imaged with automated confocal microscopy, and analyzed using a customized MATLAB script. This platform is a technical advance because it miniaturizes the assay, eliminates costly and time-consuming experimental steps, and streamlines data acquisition and analysis to enable high-throughput screening applications. As a proof of concept, this platform was used to screen a kinase inhibitor library to identify modulators of matrix alignment. A number of novel potential regulators were identified, including several receptor tyrosine kinases [c-MET, tropomyosin receptor kinase 1 (NTRK1), HER2/ERBB2] and the serine/threonine kinases protein kinase A, C, and G. The expression of these regulators was analyzed in publicly available patient datasets to examine the association between stromal gene expression and patient outcomes.

**Significance:**

ECM fiber organization and alignment contribute to metastasis in a number of cancers and are a known prognostic stromal factor; however, the mechanisms controlling matrix organization remain unclear. Here, a high-throughput assay was developed to enable discovery-based screening for an *in vitro* ECM fiber alignment assay. As proof of concept, this platform was used to screen a kinase inhibitor library and identified several novel modulators of matrix alignment.

## Introduction

The extracellular matrix (ECM) provides a structural and biochemical niche that regulates cell function and supports tissue homeostasis. During tumor progression, the normal ECM architecture undergoes dramatic remodeling. In the majority of normal mesenchymal compartments of organs that typically develop epithelial cancers (e.g., breast, lung, and pancreas), interstitial ECM fibers are randomly oriented (e.g., isotropic). However, during tumor progression, activated stromal fibroblasts [i.e., cancer-associated fibroblasts (CAF)] remodel the ECM by straightening and aligning these fibers, rendering them anisotropic (1, 2). During early breast tumor development, the ECM is organized parallel to the tumor boundary. In later stages of tumor progression, the fibers become aligned and perpendicularly oriented to the tumor edge. ECM alignment provides “tracks” for cancer cells to invade the stroma, intravasate into nearby blood vessels, and metastasize (2–6). Furthermore, recent studies have shown that this aligned matrix can block immune cell infiltration into the tumor core, highlighting the role of matrix organization in regulating the immune response to tumors (7). In breast cancer, these ECM fiber alignment phenotypes are termed “tumor-associated collagen signatures” and are separated into three types, TACS-1, 2, and 3 (2). ECM fiber alignment in tumor tissue is measured using second harmonic generation microscopic imaging to visualize collagen fiber structure within tissue sections, and fiber alignment is quantified using various image processing softwares. The presence of aligned perpendicular fibers in the tumor stroma, TACS-3, correlates with low overall and disease-free survival in patients with breast cancer (8, 9). In a recent report, increased collagen alignment also correlated with severity of breast cancer diagnosis (10). Similarly in pancreatic cancer, highly aligned ECM is a negative prognostic factor at time of pancreatic ductal adenocarcinoma (PDAC) resection (11). In colon, ovarian cancer, and other epithelial cancers, collagen organization and alignment are also associated with malignancy (12–14). While compelling evidence demonstrates a correlation between tumor stroma fiber alignment and patient outcomes, analysis of tissue fiber alignment is not currently part of standard clinical diagnoses.

ECM alignment has also been directly implicated in modulating cancer cell behavior, such as cancer cell invasion and intravasation (2, 4–6). Matrix alignment *in vivo* has been shown to facilitate premetastatic breast cancer cell invasion (2, 15, 16). *In vitro*, fibroblastic-generated matrices effectively mimic *in vivo* ECM (17). Changes in ECM topography can modulate cancer cell speed (18, 19) and directionality (4, 18–21) during invasive spread within these natural substrates. Furthermore, matrix alignment can promote cell migration even under counterintuitive conditions, including increased chemokine gradient and/or concentrations or matrix stiffness (4, 22). ECM derived from CAFs *in vitro* mimics the ECM alignment observed in the tumor microenvironment (1, 23), which effectively simulates the ECM's protumoral stimulation via regulation of cellular function (ref. 24; i.e., increased migration, invasion, and intravasation). However, disorganized, isotropic ECM derived from normal (or normalized) fibroblasts represses these tumorigenic traits (13, 18, 19).

Because of the compelling evidence supporting the role of ECM alignment in human disease, considerable effort has been devoted to uncovering the genes and pathways that regulate matrix organization and strategies for therapeutically targeting alignment. CAFs, fibroblasts in the tumor stroma that have been activated to a myofibroblast phenotype, are the principal regulators of the ECM, both by producing ECM molecules and controlling ECM organization and alignment. Therefore, CAFs are an obvious therapeutic target. However, directly eliminating the CAF population has also been shown to reduce the total amount of ECM and can promote tumor progression. For example, ablating CAFs in murine models of PDAC by targeting the myofibroblast marker α-smooth muscle actin or the sonic hedgehog signaling pathway, promoted tumor aggressiveness and decreased survival (25, 26). In addition, clinical trials therapeutically targeting CAFs were halted because of concerns for patient safety (27–29). While ablation of the ECM or CAF populations can promote tumor growth and invasion, restoration of a tumor suppressive, disorganized stroma while maintaining the fibroblast population will be a powerful strategy to inhibit cancer cell invasion and metastasis (30, 31).

Collagen crosslinking enzymes, lysyl oxidases (LOX), have also been found to be critical regulators of ECM alignment. LOX expression correlates with aligned collagen at the invasive front of ductal breast carcinomas (32). Inhibition of LOX-like 2 (LOXL2) decreased matrix alignment and tumor volume in a murine breast cancer model (6). Furthermore, inhibition of LOX with β-aminopropionitrile (BAPN) in a murine model reduced tissue stiffness and ECM alignment surrounding mammary tumors (33). However, lack of a complete crystal structure of LOX has hampered development of a specific pharmacologic inhibitor (34). In addition, in a clinical trial in which the LOX inhibitor, BAPN, was applied topically to reduce hypertrophic fibrotic scarring, the trial was halted because of toxicity issues (34).

In addition to fibroblast activation and matrix remodeling enzymes, engagement of the cell-matrix adhesion and mechanotransduction machinery in CAFs is critical for ECM organization and alignment. Several integrins, the transmembrane receptors that mediate cell-matrix adhesion, have been found to play important roles in ECM alignment, including integrins α_2_β_1_, α_v_β_3_, and α_v_β_5_ (35–38)_._ The Rho-Rho kinase (ROCK) signaling pathway, which is activated by cell-matrix adhesion, and actomyosin contractility are key regulators of ECM remodeling and alignment (13, 39–41). Indeed, cellular traction forces generated by actomyosin contractility direct FN matrix assembly (42), which is needed for collagen fibrillogenesis. However, therapeutic inhibition of the mechanotransduction machinery has remained challenging. Both FAK and integrin inhibitors have entered early clinical trials; however, FAK inhibition has demonstrated high rates of toxicity, and integrin inhibitor trials have frequently been terminated because of lack of efficacy (43, 44). ROCK is a promising target, yet despite two decades of research there is no clinically approved inhibitor (45–48). Targeting actomyosin contractility directly is challenging, as this pathway is vital for cardiovascular function, and side effects are dose limiting.

While several molecular mechanisms regulating matrix alignment have been identified and have shown remarkable success in preclinical disease models, supporting the significant role of matrix alignment in cancer, they have not translated to clinical use. This is likely due to the ubiquitous nature of several of these pathways in normal cellular function, the difficulty in developing specific pharmacologic inhibitors, and that some of these strategies ablated the fibroblastic or ECM populations, promoting tumor progression (25, 26). As an alternative approach, identifying mechanisms that normalize functional ECM topography while maintaining the fibroblast population will likely yield more tractable therapeutic targets (30, 31, 49).

There is an unmet need to identify new mechanisms and therapeutic strategies that promote ECM normalization as opposed to its elimination (30, 31). To therapeutically restore normal ECM organization with the goal of limiting cancer cell invasion and metastasis [including metastatic reseeding (50, 51)], it will be necessary to elucidate the molecular mechanisms governing ECM remodeling. To investigate ECM remodeling and fiber alignment *in vitro*, cells can be embedded in a three-dimensional collagen matrix, and collagen fiber organization visualized using confocal reflectance microscopy (21, 52, 53). This method has been used to examine how interstitial flow induces fibroblast remodeling of collagen fibers (52) and to investigate how breast cancer cells reorganize and align the ECM during invasion (21). As an alternative to providing a purified ECM, fibroblasts can be cultured to produce their own ECM *in vitro*, termed cell-derived matrices (CDM; refs. 1, 13, 20, 18). Fibroblastic CDM are the gold standard model for investigating mechanisms regulating ECM alignment *in vitro* (1, 13, 20, 18). CDM are produced by fibroblasts cultured in the presence of ascorbic acid to stabilize collagen incorporation into the ECM (54). Using this *in vitro* system, it has been shown that CAFs produce significantly more aligned ECM than normal fibroblasts, reproducing the characteristics of the *in vivo* TACS-3 and tumor microenvironment (1, 13, 20, 18). Using CDM to study fiber alignment significantly reduces the experimental cost and complexity as compared with three-dimensional collagen culture by eliminating the need for the exogenous collagen matrix; however, there is less control over the properties of that matrix (composition, density, mechanical properties) and how those properties may feedback to regulate cell behavior.

While CDM have been an essential tool for studying matrix organization in a controlled and reproducible manner *in vitro*, current methods for generating and analyzing CDM are not compatible with high-throughput screening (HTS) approaches, limiting their ability to conduct unbiased discovery-oriented screens to identify new molecular mechanisms or novel therapeutics. The assay is experimentally labor-intensive, expensive, and cumbersome for data analysis. Previous studies have miniaturized the production of CDM in a 96-well format to investigate the effects of a given type of CDM on cancer cell responses to assorted chemotherapies (55); however, analysis of matrix alignment has not been adapted for HTS applications. To address this need, we adapted a CDM alignment assay to a robust and reproducible HTS platform. The key advancements that enabled this platform include the development of a scaled-down experimental workflow for use in 384-well plates, streamlining of ECM visualization, and the development of an automated fiber alignment scoring system. The assay was credentialed for HTS, and a kinase inhibitor library was screened to demonstrate the use of the platform. This preliminary kinase inhibitor screen identified small molecules that target several known regulators of matrix alignment in addition to identifying compounds that target novel matrix alignment modifiers.

## Materials and Methods

### Cell Culture

NIH/3T3s were purchased from ATCC (catalog no. CRL-1658, RRID:CVCL_0594), cell line verification performed by ATCC, and used within 6 months of receipt. *Pten^−^^/^^−^* (*Fsp-Cre;Pten^loxP^^/^^loxP^*) murine mammary fibroblasts (MMF; strain FVB/N, female) were a gift from Dr. Michael C. Ostrowski, generated previously (56–58) in compliance with federal and University Laboratory Animal Resources regulations and approved by the Ohio State University Institutional Animal Care and Use Committee under protocol 2007A0120-R1 (PI: Michael Ostrowski). No *in vivo* mouse experiments were conducted as a part of this study. Cell line verification was not performed for the *Pten^−^^/^^−^* null MMFs. All cells were tested for *Mycoplasma* upon receipt and every 3 months using PromoKine *Mycoplasma* PCR Kit I/C (VWR, catalog no.10181-028). Cells were cultured in high-glucose DMEM (Thermo Fisher Scientific, catalog no. 11965118) supplemented with 10% FBS (VWR, catalog no. 97068-085), 2 mmol/L l-glutamine (Thermo Fisher Scientific, catalog no. 25030-081), 10 U/mL penicillin, and 10 μg/mL streptomycin (Thermo Fisher Scientific, catalog no. 15140-122). For the *Pten^−^^/^^−^* MMF, FBS was heat inactivated in a 56°C water bath for 30 minutes. Cells were maintained at 37°C and 5% CO_2_. NIH/3T3s were used from passages 35–50 and *Pten^−^^/^^−^* null MMFs from passages 30–50.

### Gelatin Coating Plates

384-well high-content imaging film bottom black microplates (Corning, catalog no. 3603) were coated with gelatin according to previously published protocols (59). Briefly, plates were incubated with 0.2% (w/v) gelatin (bovine skin; Millipore Sigma, catalog no. G9391) in PBS for 1 hour at 37°C. The plates were then washed three times in PBS and incubated with 1% glutaraldehyde [volume for volume (v/v)] (Millipore Sigma, catalog no. G6257) in PBS for 30 minutes at room temperature. The plates were then washed three times in PBS and incubated with 1 mol/L ethanolamine (Millipore Sigma, catalog no. 15014) in sterile water for 30 minutes at room temperature. The plates were washed three times in PBS and stored at 4°C until further use. For 384-well plates, 40 μL was used in each well, except for the PBS washes, where 60 μL was used per well.

### Fluorescent Labeling of Fibronectin

Fibronectin was labeled in-house with NHS-Fluorescein according to previously published protocols (60) with minor modifications. Briefly, 10 mg of bovine fibronectin (MilliporeSigma, catalog no. F4759) was suspended in PBS at 1 mg/mL. Fibronectin was dialyzed in PBS overnight using 8 kDa molecular weight cut-off dialysis tubing, then incubated with 125 μL of 1 mol/L sodium bicarbonate (Sigma-Aldrich, catalog no. S8875) in water, pH 9, and 125 μL of 1 mg/mL NHS-Fluorescein (Thermo Fisher Scientific, catalog no. PI-46409) in dimethyl sulfoxide (DMSO) for 2 hours at room temperature. Labeled fibronectin was separated using PD-10 desalting columns (GE Life Sciences, catalog no. 17085101) and the absorbance was measured at 280 and 494 nm for 50 μL of solution in a 96-well plate. An equivalent volume of PBS was used to correct for background absorbance. The values were corrected for a path length of 1 cm and the fibronectin concentration was calculated using the following formula (60):



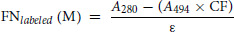



where CF is a correction factor adjusting for the amount of absorbance at 280 nm caused by the dye, in this case 0.3. ε is the molar extinction coefficient for fibronectin, in this case 677,800 M^−1^ cm^−1^.

The degree of labeling of the fibronectin was calculated as follows (60):



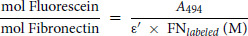



where εʹ is the molar extinction coefficient of fluorescein, in this case 68,000 M^−1^ cm^−1^.

For these experiments, the labeled fibronectin stock was at 0.3 mg/mL and was found to have a degree of labeling of approximately 6.

### Low-throughput 24-well CDM Production and Immunofluorescent Staining

CDM were produced in 24-well plates as described previously (59, 61). Briefly, NIH/3T3s were seeded on 12 mm gelatin-coated glass coverslips in 24-well plates at 75,000 cells/cm^2^ in high-glucose DMEM with 10% heat-inactivated FBS. The medium was changed 24 and 72 hours after plating and was supplemented with 50 μg/mL ascorbic acid (MilliporeSigma, catalog no. A4403), and TGFβ1 (Peprotech, catalog no. 100-21; vehicle control, 0.1, 1, 5, or 10 ng/mL). For ROCK inhibition experiments, Y-27632 (Abcam, catalog no. ab120129; DMSO control, 1 or 10 μmol/L) was added to the media 24 and 72 hours after plating. Samples were fixed 5 days postseeding by removing half of the medium from each sample and adding an equivalent volume of fixative [4% (w/v) paraformaldehyde (Electron Microscopy Sciences, catalog no. 15710) and 5% (w/v) sucrose (Thermo Fisher Scientific, catalog no. S5500) in PBS] for 30 minutes at room temperature. The samples were permeabilized in 0.5% Triton X-100 for 5 minutes and blocked with 10% normal goat serum (Invitrogen, Thermo Fisher Scientific, catalog no. 16210072) for 30 minutes before incubating with 1:200 with a rabbit anti-fibronectin antibody (Abcam, catalog no. ab23750, RRID:AB_447655) in blocking solution for 1 hour. Samples were washed three times and incubated with 1:500 AlexaFluor-conjugated goat anti-rabbit (Thermo Fisher Scientific, catalog no. A11034, RRID:AB_2576217) and 1:2,000 Hoechst 33342 (Thermo Fisher Scientific, catalog no. H3570) in blocking solution for 1 hour. The samples were washed three times before mounting on slides using ProLong Gold Antifade solution (Life Technologies, Thermo Fisher Scientific, catalog no. P36930). CDM imaging and fiber orientation analyses were performed as described previously (61). To improve data visualization, all images in this study were pseudocolored by orientation relative to the mode angle of the fibers in that image using Adobe Photoshop (RRID:SCR_014199) to shift the color scale of each individual image such that all mode angles are displayed as the same color, cyan (59).

### 384-well Fibroblast-derived Matrix Production and Drug Screening

On day 0, *Pten^−^^/^^−^* MMF were seeded in a 384-well gelatin-coated plate at 50,000 cells/cm^2^ (1,500 cells/well) using a multichannel pipet and 40 μL of medium per well. The plates were rocked gently on the lowest setting of an orbital shaker (Belly dancer, IBI Scientific) for 10 minutes at room temperature prior to transfer to the incubator to promote even seeding. On days 1 and 3, the media was replaced, and cells were treated with DMSO control or 1 or 10 μmol/L of a kinase inhibitor library (Cayman Chemical #10505, batch no. 0550547), 50 μg/mL ascorbic acid, and 6 μg/mL fluorescently labeled fibronectin (labeling performed as described above). The drug library was diluted 1:10 in DMSO, and subsequent dilutions were performed in cell culture medium such that the final medium contained 0.1% (v/v) DMSO regardless of drug concentration. 0.1% DMSO was used as a negative control and 10 μmol/L Y-27632 (a ROCK inhibitor in 0.1% DMSO) was used as a positive control. On day 5, the matrices were fixed by incubation with 40 μL/well of 4% (w/v) paraformaldehyde/5% (w/v) sucrose solution for 30 minutes. The fixative was removed, and nuclei were stained using 1:1,000 Hoechst 33342 in PBS for 1 hour, prior to washing the matrices three times in PBS. After the final wash, 40 μL of PBS was added to each well, and the plate was stored in the dark at 4°C until imaging.

### Automated Imaging of Fibroblast-derived Matrices

Matrices were imaged at room temperature using a Nikon A1R confocal microscope with a 20× air objective (N.A. 0.75) and a 4× optical zoom. The NIS Elements software was used to automate image acquisition. Z-stacks were taken through an 8 μm depth at 1 μm intervals using the microscope's perfect focus system to set the focal point of the matrix at the center of the Z-stack. Four locations were randomly generated within a 0.8 mm restricted radius and imaged in each well.

### Automated Image Analysis

Images were exported as individual Tag Image File Format (TIFFs), and a custom MATLAB (R2018a, RRID:SCR_001622) code was written to automate image analysis for quality control, fiber alignment, and nuclei number, with ImageJ (v1.50e, RRID:SCR_003070) running under the command of MATLAB using the Miji plugin (v1.3.6, downloaded from http://bigwww.epfl.ch/sage/soft/mij/; ref. 62). Briefly, images were imported into MATLAB, passed to ImageJ, and z-projected using the maximum intensity of each pixel. As a quality control for insufficient fibrillogenesis or imaging errors in each field of view (FOV), the percentage of pixels in each z-projection that fell below the threshold of the background signal [100 arbitrary units (a.u.) of fluorescence intensity] was quantified. This background level was chosen for this study based on images that had little to no matrix present and may need to be adapted for other imaging setups. Any FOV with >10% of pixels at or below the background was removed from further analysis. This cutoff was chosen by analyzing the distribution of background pixel percentages across all images in the study. In addition, wells that had two or more (of four total) FOVs flagged for exclusion were excluded from further analysis. Finally, drug treatments that resulted in the exclusion of two or more replicate wells were excluded from the final hit list. To quantify the number of nuclei per image, the Hoechst channel images were z-projected, and a Gaussian blur was performed with σ = 2. The images were thresholded and watershed segmentation was performed to separate overlapping nuclei. The number of nuclei in each image was counted and compared with the control. Wells with <40% of the nuclei of the control were considered to have high toxicity which could affect matrix production and were excluded from subsequent analysis.

To quantify fiber alignment, each stack of the fibronectin channel images was z-projected and made into a 32-bit image, and the plugin OrientationJ was used to create a histogram of the fiber alignment, which was imported back to MATLAB and stored. The histogram was centered at its mode, and the fraction of fibers within 20 degrees of the mode was calculated as a metric of the fiber alignment. The average of the four locations within each well was taken as the final alignment value for that well.

### Determination of Hits

The robust Z-score was calculated for each screen according to the following formula:



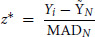



where 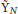 is the median value and MAD_N_ is the median of absolute deviation from the negative control (63). A composite Z-score was calculated by averaging the values from each experiment (*n* = 3), and hits were ranked according to this value.

### Stromal Gene Expression and Patient Outcomes Analysis

The correlation between gene expression in ductal breast carcinoma stroma and patient 5-year survival data for the Boersma and colleagues cohort (64) was downloaded from Oncomine (oncomine.org). To confirm the prognostic significance of the selected genes in ductal breast carcinoma samples or specifically in ductal breast carcinoma stroma, overall survival and gene expression data from The Cancer Genome Atlas (TCGA) Breast Invasive Carcinoma and Boersma ductal breast carcinoma stroma cohorts were collected from the cBio Cancer Genomics Portal (65, 66) and the Gene Expression Omnibus GSE5847 (GEO, RRID:SCR_005012). The respective cohorts were stratified into high (upper 50%) and low (lower 50%) expression groups for each gene, and Kaplan–Meier curves were generated. Differences in survival were determined by log-rank tests.

### Statistical Analysis

Statistical analyses were performed using GraphPad Prism v8 (RRID:SCR_002798). For comparisons between multiple groups, one-way ANOVA was performed, followed by Tukey multiple comparisons posttest. To determine whether the drug library used was skewed toward alignment inhibitors, a Shapiro–Wilk normality test was used on the composite robust Z-scores. Differences in patient survival were assessed using unpaired two-tailed *t* tests. Differences in patient survival curves were assessed using the Mantel–Cox log-rank test. Differences were considered statistically significant at *P* < 0.05.

### Data Availability

Robust Z-scores for the entire inhibitor screen are presented in Supplementary Table S1. The MATLAB code for data analysis is available at GitHub: https://github.com/jtsharick/HighThroughput_FDM_Screen/blob/main/HT_Matrix_Analysis_Paper_FINAL.m. Gene expression data were collected from Oncomine and GEO (GSE5847). Additional data generated in this study are available from the corresponding author upon request.

## Results

### Matrix Alignment Assay is Suitable for Adaption to HTS

**FIGURE 1:**
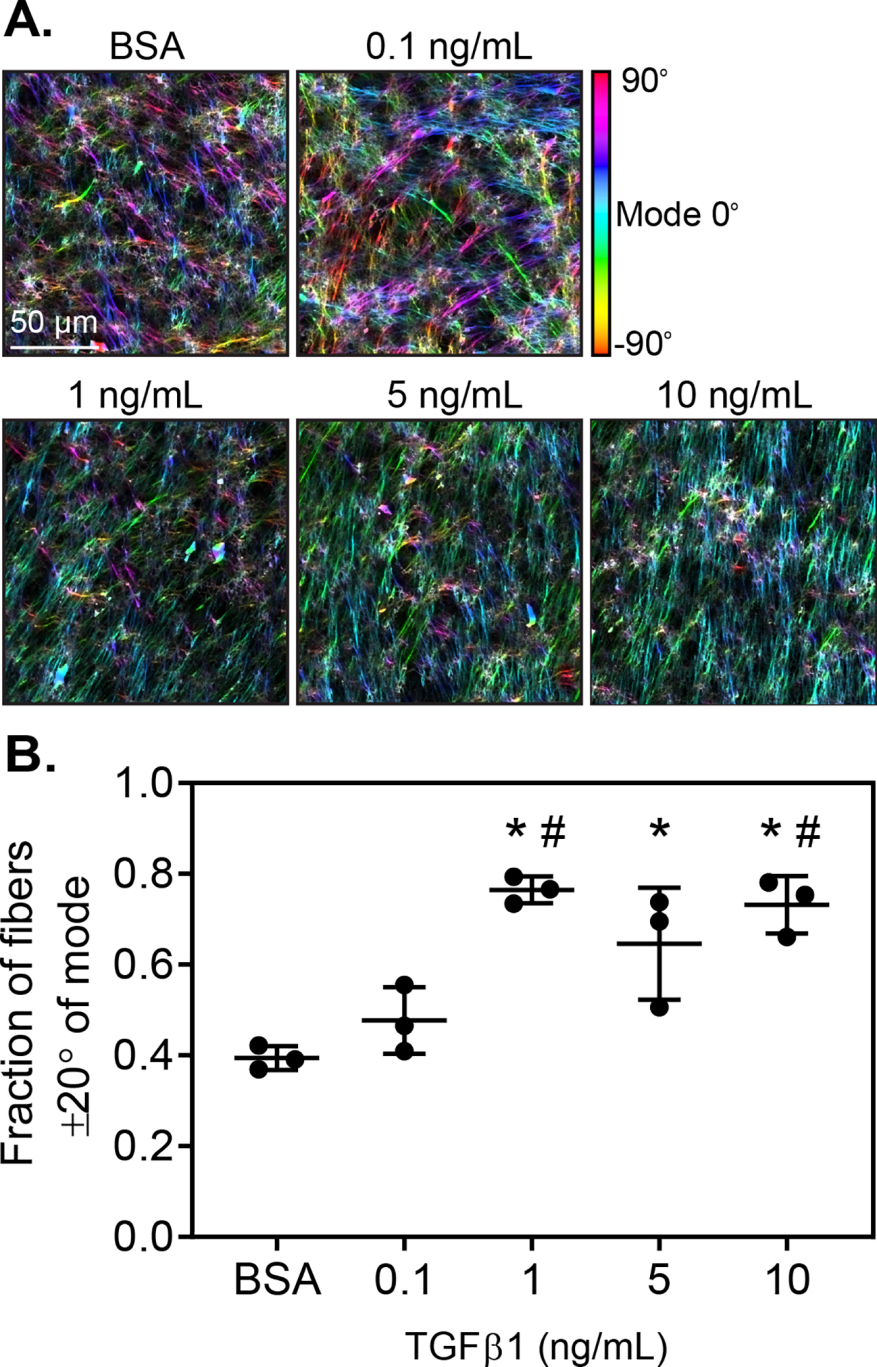
TGFβ1-induced matrix alignment. **A,** OrientationJ pseudocolor analysis of FN fiber orientation of CDM produced by NIH/3T3 fibroblasts treated with TGFβ1 or BSA control. **B,** Quantification of the fraction of fibers aligned within 20 degrees of the mode orientation. Points indicate three separate experiments. Error bars indicate SD. *, *P* < 0.05 as compared with BSA control; #, *P* < 0.05 as compared with 0.01 ng/mL TGFβ1.

CDM faithfully recapitulate the organizational characteristics of the *in vivo* stroma from which the fibroblasts originated (13, 35, 67). Specifically, the CDM produced by CAFs are highly organized and aligned parallel (i.e., anisotropic), whereas the CDM produced by fibroblasts isolated from normal tissue are disorganized (i.e., isotropic). However, the inherent heterogeneity of primary cell cultures and the limited time in culture are significant limitations for HTS design and execution. Adaptation of the CDM assay to work with well-established immortalized cell lines is a key step toward the development of an HT CDM platform. To this end, we selected the mouse embryonic fibroblast cell line NIH/3T3 and tested its compatibility with the CDM assay, as it is one of the most widely used fibroblast cell lines in biological studies and amenable to expansion for genetic and compound HTS campaigns. Using the low-throughput 24-well CDM assay as an initial step to examine the suitability of the assay for HTS, NIH/3T3 cells were stimulated with 0.1 to 10 ng/mL of TGFβ1 to induce CDM alignment to a similar degree as that observed in diseased tissue or with CAFs (refs. 1, 13, 20, 18; Fig. 1). TGFβ is a critical factor responsible for fibroblast activation in cancer (68, 69) and matrix alignment (70). Furthermore, in previous reports, TGFβ1-treated fibroblasts significantly increased collagen alignment *in vivo* in a murine head and neck squamous cell carcinoma xenograft model, whereas control fibroblasts had no effect on alignment (70). Consistent with these studies, NIH/3T3s treated with 1–10 ng/mL TGFβ1 produced highly aligned matrices, as indicated by the increased fraction of fibers with an alignment angle within 20 degrees of the mode angle, as compared with the control cells (Fig. 1). To examine the suitability of the CDM for HTS, the Z’-factor, which indicates the degree of separation of the positive and negative signals within the assay, and the coefficient of variance were determined. For the control and 1 ng/mL TGFβ1-treated matrices, the Z’-factor was greater than 0.5, and the coefficient of variation was 3%–4%, indicating sufficient separation of the positive and negative signals, low variance, and feasibility for adaptation to HTS (71).

### Miniaturization and Streamlining of the Matrix Organization Assay for a HTS Platform

**FIGURE 2:**
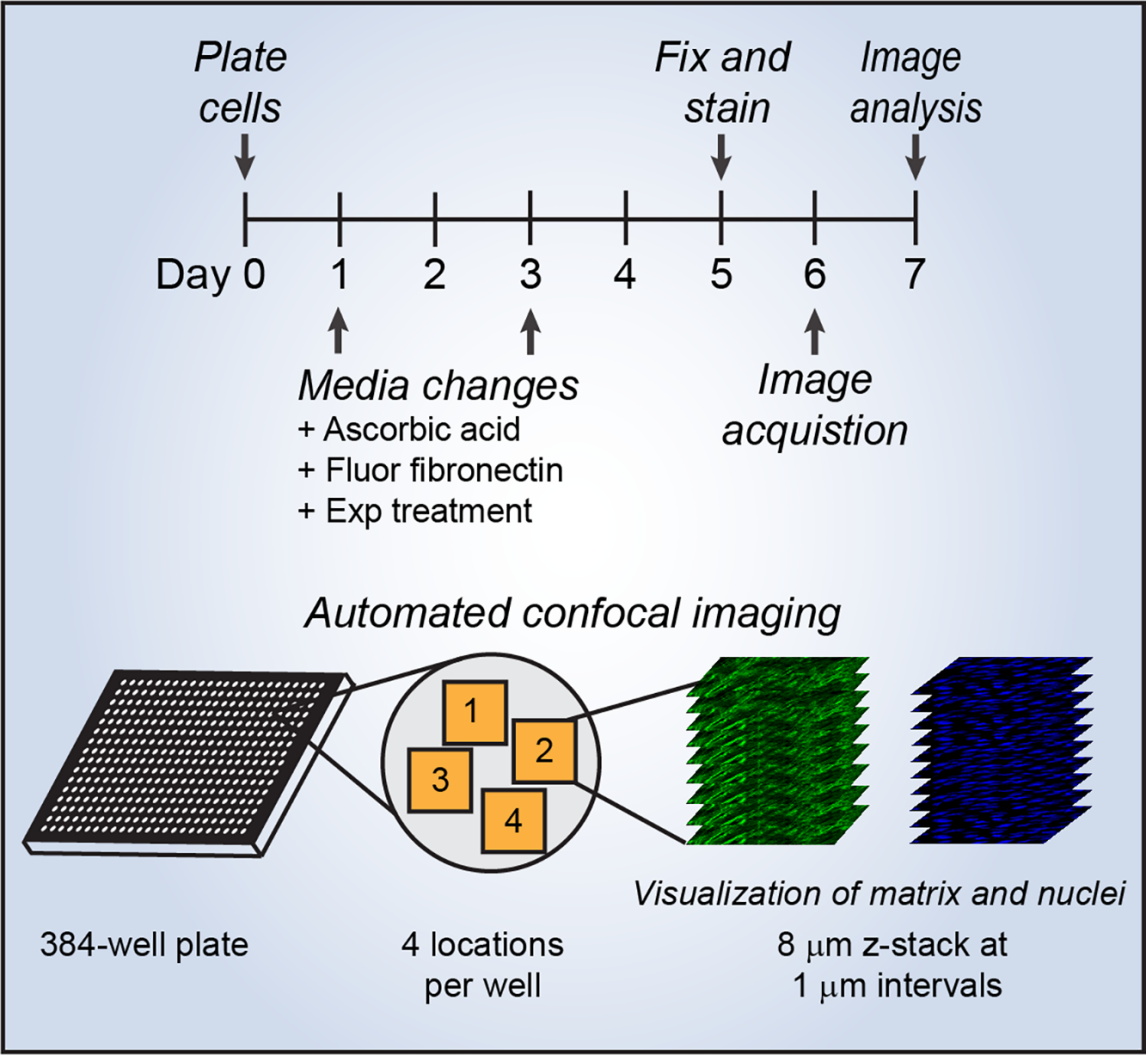
Schematic overview of experimental setup for high-throughput matrix assay. Fibroblasts were plated on day 0 in gelatin-coated 384-well plates. The media was changed on days 1 and 3 with the addition of ascorbic acid, fluorescently labeled fibronectin, and experimental treatments. On day 5, the samples were fixed, and the nuclei stained. Automated confocal imaging was used to acquire images of the samples in the plate, with 8 μm Z-stacks taken at four positions within each well.

To develop a HTS screening platform for the CDM alignment assay, the assay was miniaturized to a 384-well format. Black film bottom microplates were used to provide a flat, thin surface necessary for high content imaging (Fig. 2). The plates were coated with gelatin prior to cell seeding to promote CDM adhesion to the well and prevent detachment during media changes and processing steps. After addition of the cells to the plate, gentle agitation at the slowest speed on an orbital shaker for ten minutes at room temperature ensured even cell seeding within the wells. The fiber organization of CDM is typically visualized through immunofluorescent labeling of fibronectin or collagen. To eliminate these costly and time-consuming immunostaining steps and to enable visualization of the matrix for the screening platform, cells were cultured with exogenous fluorescein-labeled fibronectin. Previous studies have demonstrated the incorporation of fluorescent fibronectin into CDMs (72, 73), and the addition of exogenous fluorescent fibronectin has previously been used to study fibronectin fibrillogenesis (60, 74). Furthermore, fibronectin is oriented in an equivalent manner as collagen and is the most prevalent protein expressed in CDM (1). After matrix production for 5 days, the CDM were fixed and the nuclei were stained. Images of fluorescently labeled fibronectin within the CDM and cellular nuclei were acquired using a confocal microscope equipped with an automated stage. Nikon NIS Elements software was used to select four random, nonoverlapping locations within each well for imaging. Nikon's Perfect Focus System was used to set the focal point of the matrix at the center of the Z-stack, and images of fibronectin and nuclei were captured at 1 μm intervals for a total depth of 8 μm.

**FIGURE 3:**
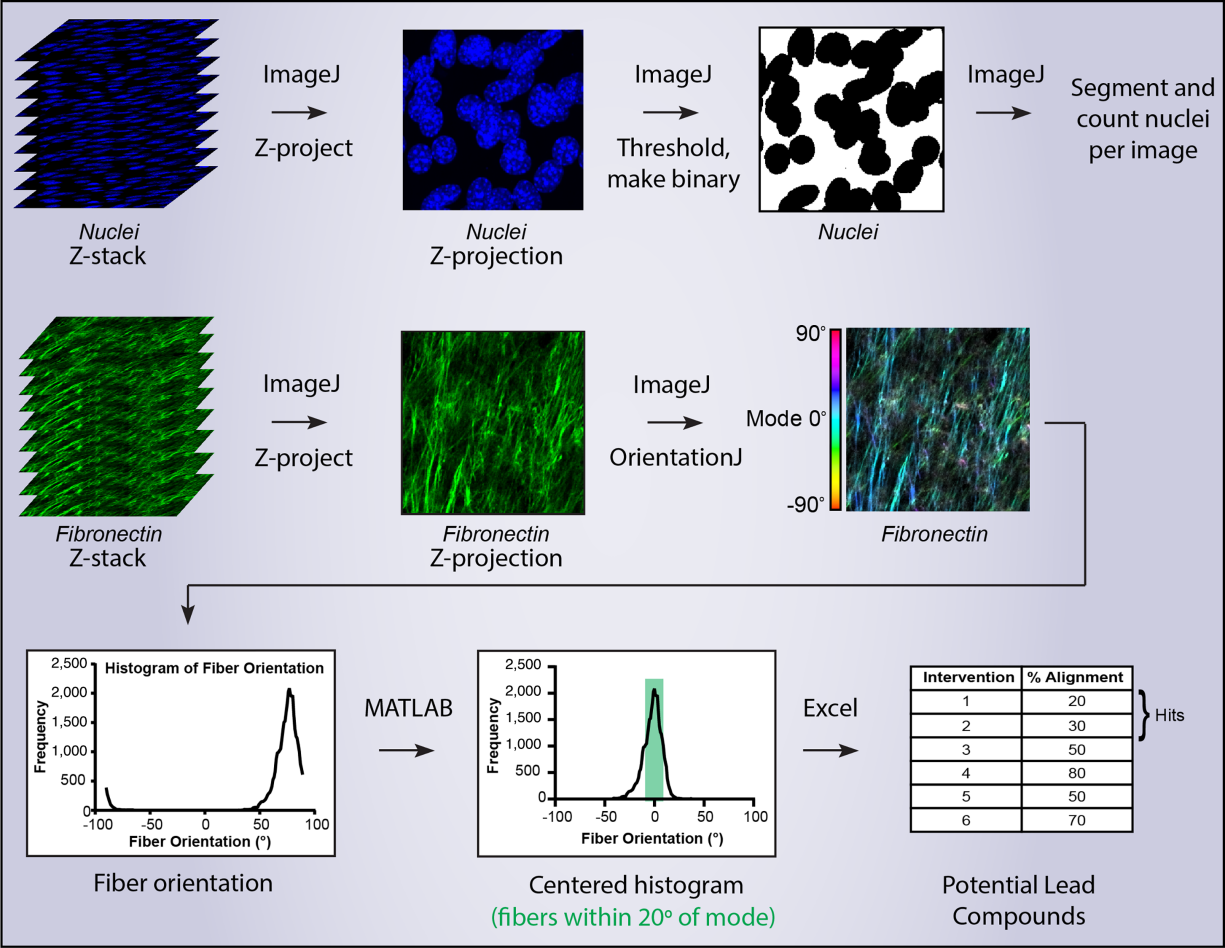
Data analysis pipeline. Image analysis was automated using a MATLAB script. ImageJ/FIJI was run under the control of MATLAB using the Miji plugin. Images were imported to MATLAB, passed to ImageJ, and Z-projections of the nuclei channel were thresholded, made binary, segmented, and counted. Z-projections of the fibronectin channel were analyzed using OrientationJ, which outputs a histogram of fiber orientation. This output is passed back to MATLAB and the histogram is centered at the mode. The fraction of fibers within 20 degrees of the mode was determined as a metric of matrix alignment.

In addition to the experimental hurdles limiting the development of a CDM alignment screening platform, the analysis in these studies is time and labor intensive. Typically, CDM organization is qualitatively and quantitatively assessed by processing multiple confocal image Z-stacks per condition with the ImageJ plugin OrientationJ (75). OrientationJ produces a pseudocolored map of the relative fiber orientations and renders a histogram of the fiber orientation (Fig. 3). The extent of CDM alignment is indicated by the fraction of fibers distributed around the mode angle, as illustrated by the highlighted region in the histogram (Fig. 3). However, further data processing is needed to center the histogram and produce a quantitative fraction of the total fibers aligned within a certain degree (typically 10 or 20 degrees) of the mode angle.

To automate image and data analysis for quality control, fiber alignment, and nuclei number, a MATLAB code was written (Fig. 3), with ImageJ running under the command of MATLAB using the Miji plugin (Supplementary Data S1; ref. 62). Confocal microscopic images were exported as individual TIFFs and the image files were imported into MATLAB and passed to ImageJ. The stack was z-projected and made into a 32-bit image, and the plugin OrientationJ was used to create a histogram of the fiber alignment, which was imported back to MATLAB and stored. To automate the data analysis process, the MATLAB code identifies the mode of the orientation angle (the angle that appears the most often in the fiber distribution), centers the histogram at the mode, assigns the mode to “0,” calculates the percentage fraction of fibers within 20 degrees of the mode to provide a quantitative measure of fiber alignment, and exports the data to a table in Microsoft Excel. The average of 3–4 locations within each well was taken as the final value of alignment for that well. To quantify the number of nuclei per image, the Hoechst channel images were z-projected, and a Gaussian blur was performed with σ = 2. The images were thresholded and watershed segmentation was performed to separate overlapping nuclei. The number of nuclei in each image was counted and compared with the control, for a final metric of percent of control nuclei (Fig. 3).The custom MATLAB code is available on GitHub: https://github.com/jtsharick/HighThroughput_FDM_Screen/blob/main/HT_Matrix_Analysis_Paper_FINAL.m

### Kinase Inhibitor Screen Identifies Novel Regulators of Matrix Alignment

To test the discovery potential of the newly developed screening platform, we analyzed a commercially available library of 154 small-molecule kinase inhibitors (Cayman Chemical). This library is enriched in compounds targeting lipid, receptor, and non-receptor tyrosine and serine kinases, and targets more than 70 protein kinase families. Kinases are involved in a variety of signaling pathways known to affect matrix alignment, including cellular contractility (61, 76), matrix adhesion (35), and fibroblast activation (77). In addition, kinases constitute nearly a quarter of the Lipinski-druggable genome (78), making them readily targetable by pharmacological inhibitors. We predicted that screening this library would identify compounds targeting known and novel regulators of matrix alignment.

*Pten* null murine fibroblasts were selected as the screen cells for this assay based on prior work, which demonstrated that these cells produce an aligned matrix *in vitro* and *in vivo* (61). Low PTEN expression in stromal fibroblasts has been associated with poor outcomes in multiple human tumors, including breast, prostate, pancreatic, and endometrial cancers (56, 58, 79–84). *In vivo* ablation of *Pten* (*Fsp-Cre;Pten^loxP/loxP^*) in fibroblasts of mouse mammary tissue led to increased collagen deposition, collagen alignment, and tumorigenesis in the presence of the Neu oncogene (56, 61). *In vitro*, CDM produced by *Pten* null *(Pten^−^^/^^−^)* murine mammary fibroblasts results in a highly organized, aligned matrix compared with the largely disorganized matrix produced by *Pten* intact (wild-type*)* fibroblasts (61, 73). As such, these cells are well suited for screening compounds that can “normalize” a highly aligned matrix.

#### Assay Performance

**FIGURE 4:**
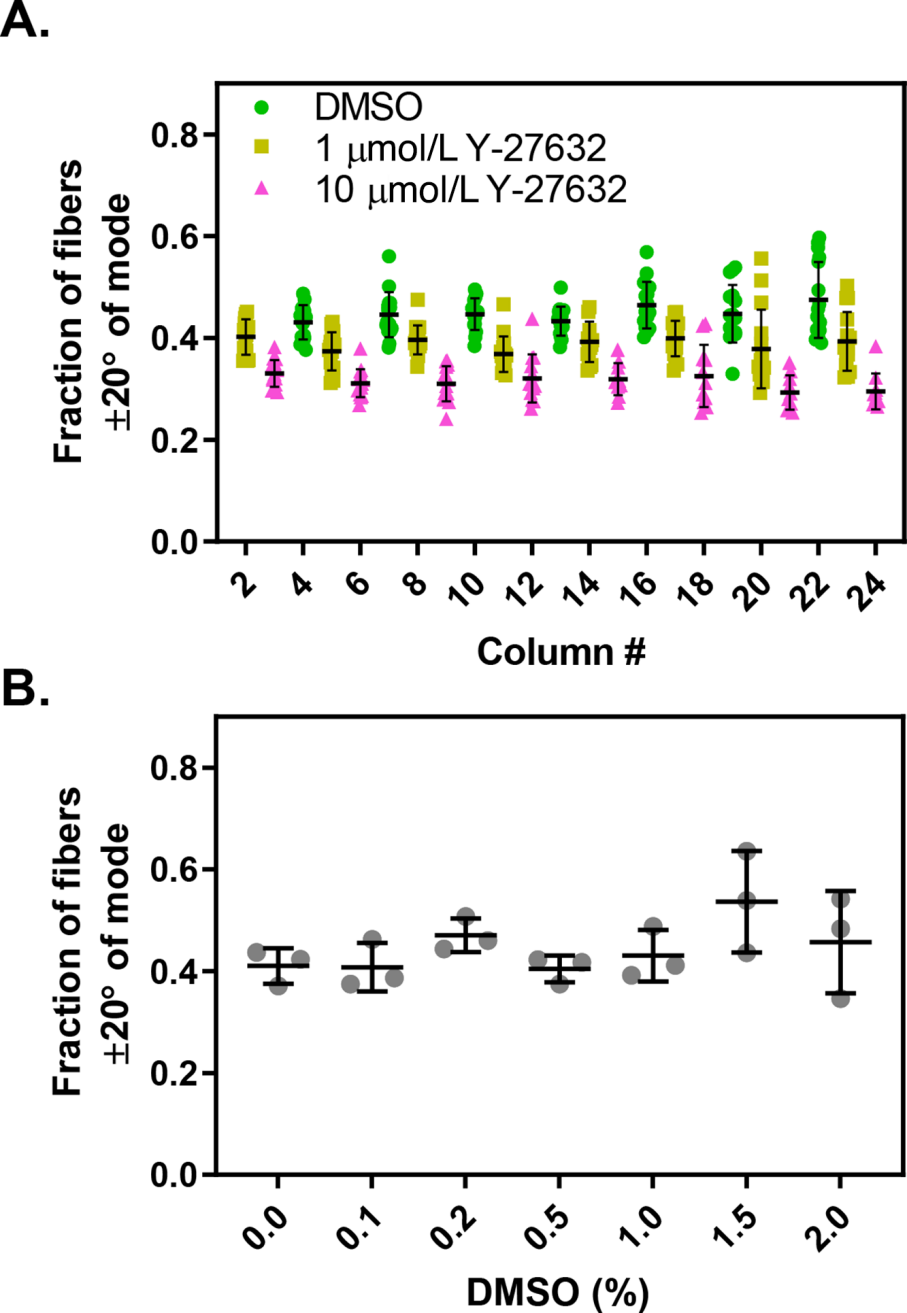
High-throughput assay characterization. **A,** Fiber alignment of fibroblasts seeded in 384-well plates and treated with Y-27632 (1 or 10 μmol/L) or control (DMSO) in an interleaved format. **B,** Fiber alignment with a range of DMSO treatment up to 2%. No significant differences.

To ensure that the assay results were unaffected by solvent or plate positioning artifacts, *Pten^−^^/^^−^* murine fibroblasts were treated with the DMSO control or two different concentrations (1 and 10 μmol/L) of Y-27632, a ROCK inhibitor previously shown to significantly reduce matrix alignment in *Pten^−^^/^^−^* fibroblasts (61), in a single column across an entire 384-well plate. Matrix alignment was assessed by determining the fraction of fibers falling within 20 degrees of the mode matrix orientation. No significant differences were observed in the average values of any column with the same treatment (Fig. 4A). However, a dose-dependent decrease in matrix alignment with Y-27632 treatment was observed, indicating that the assay can reliably measure the effects of drug treatment on matrix alignment independent of plate position. Next, the effects of DMSO concentration, a common library solvent, on matrix alignment were examined. No significant differences in alignment were found across the 0%–2% range tested (Fig. 4B).

#### Assay Execution

*Pten^−^^/^^−^* fibroblasts were plated in 384-well plates on day 0 at 5 × 10^4^ cells per well (to assure cells were confluent the next day), and the cell culture medium was replaced on days 1 and 3 to include ascorbic acid, fluorescently labeled fibronectin, and the kinase inhibitors or vehicle control. DMSO was used as a vehicle control at a final concentration of 0.1% and 10 μmol/L Y-27632 was used as a positive control to reduce matrix alignment. Y-27632 was used as a positive control for a reduction in matrix alignment, to mimic the disorganized matrix produced by normal cells as compared with the aligned matrices produced by CAFs (1, 18, 35). A total of 154 inhibitors in the Cayman kinase inhibitor library were screened at concentrations of 1 and 10 μmol/L. On day 5, the samples were fixed, and the nuclei were stained. Confocal images of the CDM and nuclei were obtained using the automated imaging setup described above. The screen was repeated on three separate days.

**FIGURE 5:**
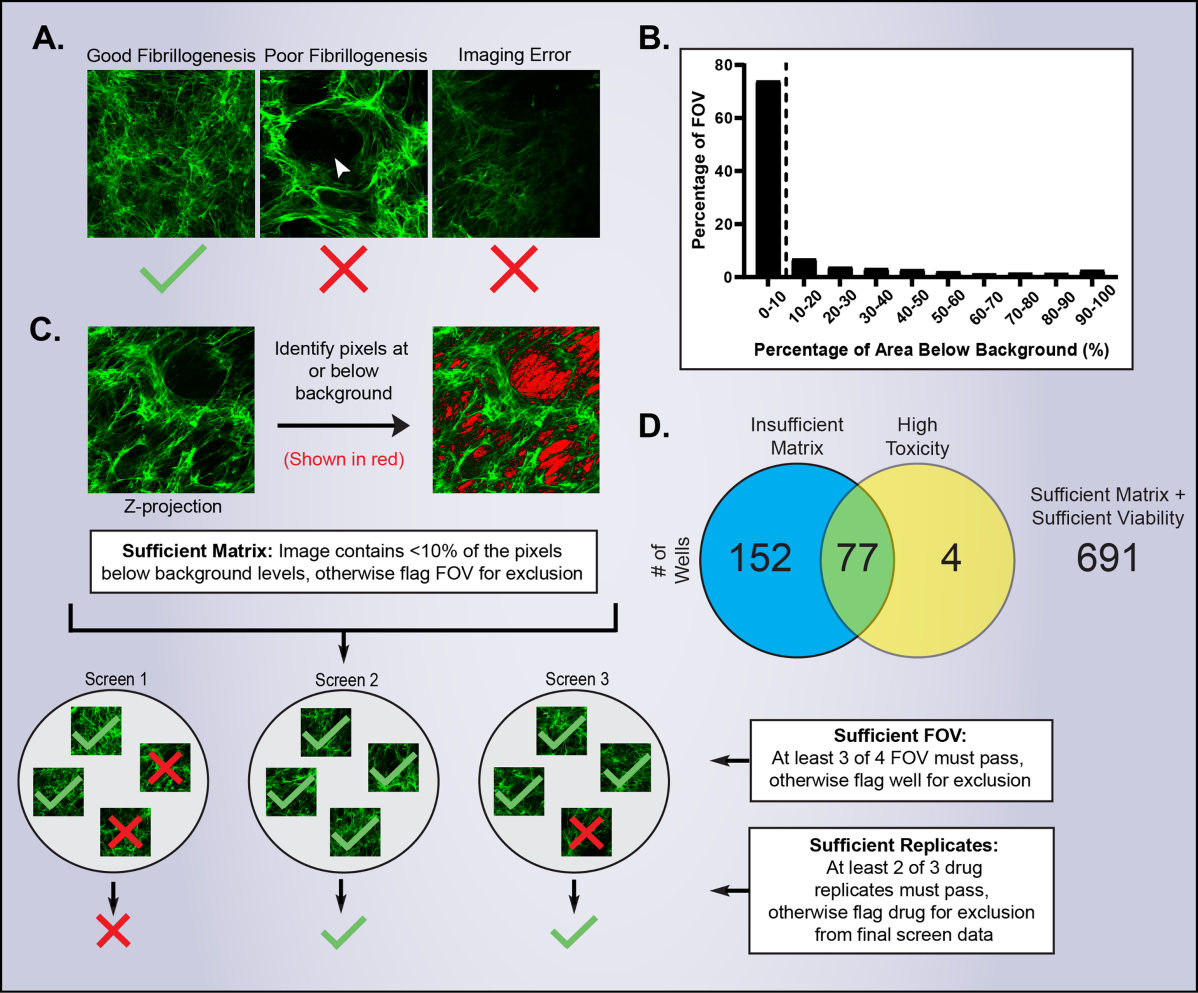
Quality control pipeline. **A,** Images from this study depicting examples of sufficient fibrillogenesis, poor fibrillogenesis, and imaging error. White arrow indicates a hole in the matrix, which signifies poor fibrillogenesis. **B,** Histogram of percentage of pixels below background for every FOV imaged in this study (*n* = 4,336). **C,** To ensure sufficient fibrillogenesis and imaging quality for alignment analysis, the fraction of pixels in the fibronectin channel that fall below background noise levels (100 a.u) is quantified for each FOV. Those with >10% of pixels below background were flagged and excluded from subsequent analysis. Drug screen wells containing less than three valid FOV were excluded from subsequent analysis, and drugs with 0 or 1 valid wells across three replicate plates were also excluded. Images in **C** show FDM treated with 1μmol/L of PP-242 (hit #13 in kinase inhibitor screen). **D,** Of 924 total wells analyzed in this study, 152 were excluded for insufficient matrix, four were flagged for high toxicity, and another 77 were flagged for both conditions.

The purpose of the HT CDM platform is to identify regulators of matrix organization; therefore, sufficient matrix must be present to measure matrix alignment. Toward this goal, wells with insufficient matrix, due to factors such as cell toxicity, deficient ECM production or fibrillogenesis, or imaging errors, were identified and excluded from further analysis (Fig. 5). Wells with insufficient matrix were defined as fields where the image contained more than 10% of pixels at the “background” level, where the background level in this study was defined as a region having an intensity level of 100 a.u. or less. The background level was determined by measuring the signal in portions of the imaged FOV that did not contain any matrix. Images that did not meet this threshold often lacked a matrix, large holes in the matrix, or imaging-related errors (e.g., out of focus, misalignment between the objective and matrix; Fig. 5A). Using these exclusion criteria, greater than 75% of the images contained sufficient matrix for further analysis of matrix alignment (Fig. 5B). Four FOV were imaged per well and at least three FOV were required to pass the above exclusion criteria for further matrix alignment analysis in that well. In addition to sufficient matrix, wells with high cell toxicity were excluded if the nuclei count was less than 40% of the control. On the basis of these exclusion criteria for insufficient matrix and cell toxicity, 229 of 924 total wells from all three screens were excluded from further analysis, with 152 wells excluded for insufficient matrix, 4 wells excluded for cell toxicity, and 77 that failed both criteria (Fig. 5D). Of the 288 excluded wells, 78% were from the conditions with the 10 μmol/L concentration of the kinase inhibitor.

**FIGURE 6:**
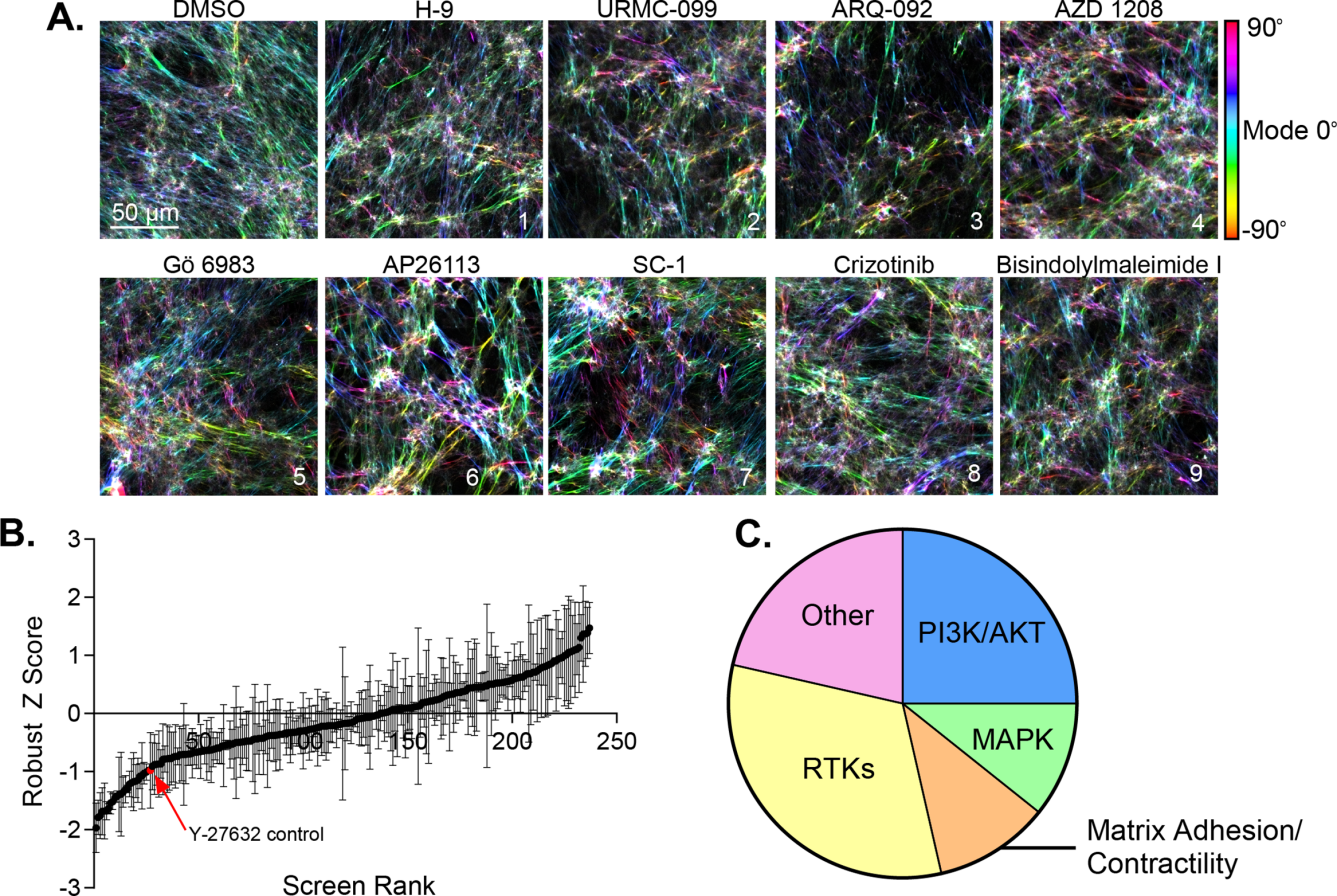
High-throughput kinase inhibitor screen results. **A,** Representative images of the negative control (DMSO) and top nine hits identified by the screen according to robust Z-score. Each image is pseudocolored by orientation relative to the mode angle. **B,** Distribution of the inhibitors in the screen ranked by robust Z-score (mean ± SEM). **C,** Pie chart of drug types represented in the 27 hits identified in this screen.

Matrix alignment was quantified using the custom MATLAB script to determine the fraction of fibers falling within 20 degrees of the mode orientation angle of the matrix. A robust Z-score was calculated from the average matrix alignment across the FOV for each condition in the screen. The robust Z-scores were then averaged across two or three screens to determine the hit ranking, excluding conditions that did not have sufficient FOV in two or three screens. The results from the screen were ranked according to the average robust Z-score and included compounds that promoted (Z > 0, where 0 represents the DMSO control, *n* = 109), and which suppressed alignment (Z < 0, *n* = 212), although the distribution was significantly skewed with more inhibitors reducing alignment (Fig. 6; Supplementary Fig. S1; Supplementary Table S1). This skew is likely library specific and may change with the composition of each library. The S-curves of the robust Z-scores from each of the three trials showed similar results (Supplementary Fig. S2). Compounds were considered hits if the average robust Z-score was less than or equal to the known inhibitor Y-27632 (1 μm), resulting in 27 compounds and a hit rate of approximately 8% (Fig. 6A; Table 1). Y-27632 was selected as the cutoff to identify hits as previous studies using the PTEN null model demonstrated that inhibition of ROCK with Y-27632 treatment reduced matrix alignment by approximately half (61). This reduction in matrix alignment was similar to the alignment measured in matrix produced by wild-type cells with intact PTEN. Furthermore, this amount of change in matrix alignment between PTEN null and wild-type cells (a 50% reduction in matrix alignment) is biologically important, as it resulted in significant changes in cancer cell morphology and migration *in vitro*. In addition, a 50% reduction in matrix alignment is similar in magnitude to the differences reported between normal cells and CAFs in the literature (1, 18, 35). The compounds did not need to meet this criterion at both concentrations (1 and 10 μm) to be considered a potential lead compound.

**TABLE 1:** Top inhibitors from the matrix alignment screen ranked by robust Z-score

Rank	Drug [Concentration]	Robust Z-Score	Protein target(s)
1	H-9 [10 μmol/L]	−1.97	PKG and PKA
2	URMC-099 [1 μmol/L]	−1.79	LRRK2, MLKs
3	ARQ-092 [10 μmol/L]	−1.76	PKB/Akt
4	AZD 1208 [1 μmol/L]	−1.68	PIMs
5	Gö 6983 [10 μmol/L]	−1.68	PKCs
6	AP26113 [10 μmol/L]	−1.67	ALK
7	SC-1 [10 μmol/L]	−1.63	ERK1
8	(R)-Crizotinib [1 μmol/L]	−1.54	c-Met, ALK
9	Bisindolylmaleimide I [10 μmol/L]	−1.52	PKCs
10	KW 2449 [1 μmol/L]	−1.45	FLT3, ABL
11	AG-825 [10 μmol/L]	−1.43	ErbB2
12	Kenpaullone [10 μmol/L]	−1.39	GSK3B, CDKs
13	PP242 [1 μmol/L]	−1.39	mTOR
14	LOXO-101 [1 μmol/L]	−1.35	Trk family
15	ML-9 [10 μmol/L]	−1.30	Multikinase
16	AZ191 [1 μmol/L]	−1.30	DYRK1b
17	IKKε [1 μmol/L]	−1.21	IKKε
18	(R)-Roscovitine [1 μmol/L]	−1.19	CDKs
19	Sorafenib [1 μmol/L]	−1.17	Raf-1
20	SB 202190 [10 μmol/L]	−1.16	p38 MAPK
21	NVP-AEW541 [1 μmol/L]	−1.15	IGF-1R
22	Tie2 Kinase Inhibitor [1 μmol/L]	−1.08	Tie2
23	PF-06463922 [10 μmol/L]	−1.07	ALK
24	ARQ-092 [1 μmol/L]	−1.03	PKB/Akt
25	PF-562271 [1 μmol/L]	−1.01	FAK
26	Torin 1 [1 μmol/L]	−0.98	mTOR
27	Y-27632 [1 μmol/L]	−0.98	ROCK-1

### Kinase Inhibitor Screen Identifies Novel Regulators of Matrix Alignment

The hits identified from the screen fell into several broad categories, as depicted in Fig. 6C. Many of the top hits were related to PI3K and protein kinase B (PKB)/Akt signaling, including inhibitors targeting Akt (ARQ-092), GSK3β (kenpaullone), mTOR (PP242 and Torin 1), and PIM kinases (AZD 1208). MAPK signaling inhibitors were also among the top hits, including compounds targeting mitogen-linked kinases (URMC-099), ERK-1 (SC-1), Raf-1 (sorafenib), and p38 MAPK (SB 202190). Identification of these pathways is likely due to the use of *Pten^−^^/^^−^* fibroblasts, as PTEN is a known regulator of PI3K/Akt and MAPK signaling. In addition, the screen identified several inhibitors that target known modulators of matrix alignment related to cell-matrix adhesion and actomyosin contractility, including myosin light chain kinase (ML-9), focal adhesion kinase (PF-562271), and Rho kinase (Y-27632). Eight identified inhibitors, approximately 30% of the top hits, targeted receptor tyrosine kinases, including c-Met [(R)-crizotinib], FLT3 (KW 2449), ErbB2 (AG-825), tropomyosin receptor kinases (LOXO-101), and Tie2 kinase (Tie2 kinase inhibitor). Inhibitors of receptors may be common hits due to the possible activation of many downstream pathways, including PI3K/Akt and MAPK signaling, and thus a higher likelihood of impinging on critical pathways related to matrix alignment. Notably, three small molecules that inhibit anaplastic lymphoma kinase (ALK), a member of the insulin receptor superfamily, were identified [AP26113, (R)-crizotinib, and PF-06463922], as well as the insulin-like growth factor 1 receptor (IGF-1R) inhibitor NVP-AEW541. ALK and IGF-1R activate the MAPK and PI3K/AKT pathways (85). IGF-1 has been shown to promote lung fibroblast activation and collagen synthesis (86, 87), and therefore may play a similar role in the tumor microenvironment.

While many of the identified hits may have been hypothesized prior to the screen, we classified about one quarter of the hits as “other” that were related to novel pathways not previously connected to matrix alignment. The top hit was H-9, an inhibitor of multiple protein kinase G (PKG) and protein kinase A (PKA) isoforms. Interestingly, PKG and PKA have previously been identified as antifibrotics in cardiac fibroblasts, antagonizing TGFβ signaling and reducing collagen production, fibroblast activation, and contractility (88–94). Other high-ranking hits included two inhibitors targeting protein kinase C (PKC) isoforms (Gö 6983 and bisindolylmaleimide I), two inhibitors of cyclin-dependent kinases [CDK; kenpaullone and (R)-roscovotine], AZ191, which inhibits dual-specificity tyrosine-phosphorylation-regulated kinase 1 B (DYRK1b), and an I-kappa-B kinase epsilon (IKKε) inhibitor.

### Screen Hits Correlate with Patient Survival

The HT *in vitro* matrix alignment kinase inhibitor screen suggested several novel regulators of matrix alignment. However, selecting the pathways to pursue further investigation and possible translational potential is not straightforward. One way to further narrow down these potential pathways is to use gene expression data from patient tumor samples to correlate pathways identified from the screen with patient outcomes.

Commonly utilized patient datasets, such as TCGA and METABRIC, focus on gene expression in tumor cells, and relatively few datasets exist that specifically examine the association between stromal gene expression and patient survival. However, several groups have generated datasets from patients with breast cancer, where the stroma surrounding the tumor was isolated using laser capture microdissection, and gene expression specifically in the stromal compartment was determined via microarrays (64, 95). Using the dataset from Boersma and colleagues (64), we correlated the expression of the predicted gene targets from the hits of the inhibitor screen with breast cancer patient outcomes. The Boersma study investigated gene signatures of inflammatory breast cancer (15 patients) and invasive, noninflammatory breast cancer (35 patients) with poor disease outcomes (less than 5-year survival). The average overall survival for patients above and below the median gene expression was compared using a *t* test during a 5-year follow-up period. Several genes appeared highly associated with survival including *AKT1, PRKCA, MET, ERBB2, NTRK1*, and *MYLK3* (genes with significant changes in Table 2, complete analysis in Supplementary Table S2). *ERBB2* has previously been identified as a critical mediator of fibrosis in several different systems (96–99). Neurotrophins and their receptors (NTRK) are expressed in fibroblasts and have been found to play a role in dermal fibroblast activation to myofibroblasts and contribute to fibrosis in a number of tissues (100, 101).

**TABLE 2:** Association of mRNA expression of related inhibitor protein targets with patient survival. Gene expression in ductal breast carcinoma stroma was correlated with patient survival at 5 years. Patients were grouped according to their survival status and gene expression was compared by *t* test. Fold change represents average gene expression in patients reported as deceased at 5 years relative to those alive at 5 years. Significant changes are highlighted in bold

Rank	Drug [Concentration]	Robust Z-Score	Protein target	Gene name	Reporter ID	5 Year *t* Test	Survival P-value	Q-value	Fold change
3	ARQ-092 [10 μmol/L]	−1.76	PKB/Akt	***AKT1***	207163_s_at	1.895	**0.033**	1.460	1.13
				*AKT2*	203808_at	0.650	0.260	1.015	1.02
				*AKT3*	212607_at	0.500	0.311	1.001	1.07
5	Gö 6983 [10 μmol/L]	−1.68	PKCs	***PRKCA***	215194_at	1.874	**0.035**	1.450	1.04
				*PRKCB*	209685_s_at	−1.296	0.893	0.978	−1.27
				*PRKCG*	206270_at	−1.204	0.880	0.976	−1.04
				*PRKCD*	202545_at	−1.180	0.876	0.975	−1.12
8	(R)-Crizotinib [1 μmol/L]	−1.54	c-Met,	***MET***	203510_at	1.811	**0.040**	1.402	1.42
			ALK	*ALK*	208211_s_at	0.854	0.200	1.064	1.03
9	Bisindolylmaleimide I [10 μmol/L]	−1.52	PKCs	***PRKCA***	215194_at	1.874	**0.035**	1.450	1.04
				*PRKCB*	209685_s_at	−1.296	0.893	0.978	−1.27
				*PRKCG*	206270_at	−1.204	0.880	0.976	−1.04
				*PRKCD*	202545_at	−1.180	0.876	0.975	−1.12
				*PRKCE*	206248_at	0.652	0.260	1.015	1.03
11	AG-825 [10 μmol/L]	−1.43	ErbB2	***ERBB2***	210930_s_at	1.781	**0.042**	1.407	1.05
14	LOXO-101 [1 μmol/L]	−1.35	Trk family	***NTRK1***	208605_s_at	2.619	**0.006**	1.905	1.06
15	ML-9 [10 μmol/L]	−1.30	Multi	***AKT1***	207163_s_at	1.895	**0.033**	1.460	1.13
			kinase	*AKT2*	203808_at	0.650	0.260	1.015	1.02
				*AKT3*	212607_at	0.500	0.311	1.001	1.07
				*MYLK1*	not in database
				*MYLK2*	not in database
				***MYLK3***	217623_at	2.067	**0.025**	1.533	1.03
				MYLK4	not in database
24	ARQ-092 [1 μmol/L]	−1.03	PKB/Akt	***AKT1***	207163_s_at	1.895	**0.033**	1.460	1.13
				*AKT2*	203808_at	0.650	0.260	1.015	1.02
				*AKT3*	212607_at	0.500	0.311	1.001	1.07

**FIGURE 7:**
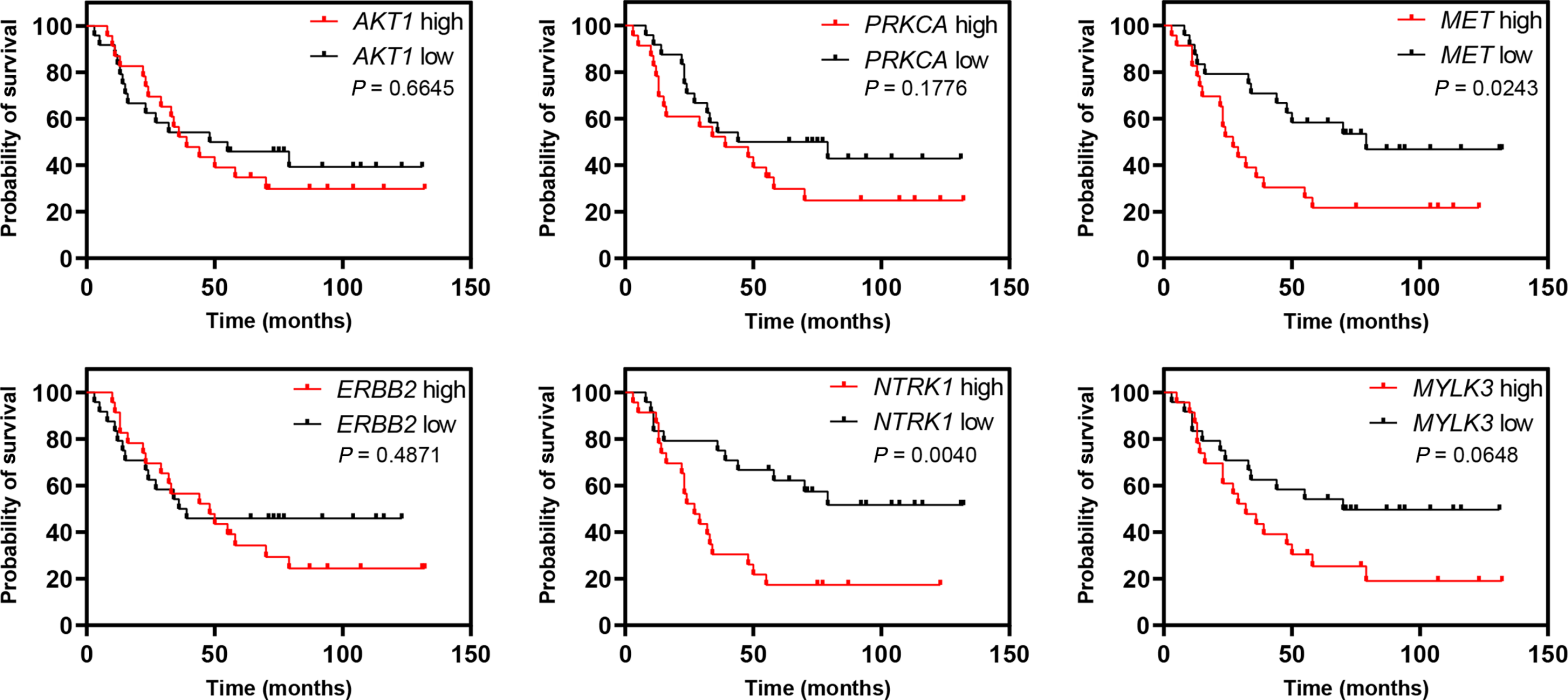
Kaplan–Meier curves for recurrence-free survival. Patients from the Boersma cohort were stratified according to gene expression at the median gene expression. *P* values were calculated by the log-rank test.

The prognostic significance of these six genes was then evaluated more rigorously using the Kaplan–Meier analysis (Fig. 7). For each gene, the patient population was stratified according to high or low expression levels along the median. The resulting curves were statistically compared using the log-rank test. High expression of *MET* and *NTRK1* were associated with a significant decrease in patient survival. *MYLK3* was not significant at α = 0.05; however, it showed a clear trend of decreased patient survival with high expression (*P* ∼ 0.07). A similar Kaplan–Meier analysis was performed for these genes using data from TCGA invasive breast carcinoma cohort (Supplementary Fig. S3). These analyses showed similar trends to the analyses of the breast cancer stroma samples but were not as strongly prognostic.

## Discussion

Expression of several genes whose protein products are inhibited by hits from this screen appeared to be associated with poor patient outcome. c-MET is a receptor tyrosine kinase that is commonly upregulated in a wide variety of carcinomas and is activated in response to paracrine hepatocyte growth factor signaling from the stroma (102–104). However, whether c-MET is upregulated in the stroma of breast cancer is unclear. PKC is likewise upregulated in a variety of different carcinomas, but has eight different isozymes (α, βI, βII, γ, δ, ε, θ, and η) whose individual contributions remain unclear (105). Interestingly, several PKC isozymes have been shown to play a role in human dermal fibroblast activation by TGFβ (106), suggesting that PKC may contribute to an activated CAF phenotype. PKCα was chosen for its association with patient survival in this study as it is most strongly inhibited by each of the inhibitors found as hits in this screen, although other isozymes may also play a role. A limiting factor of the analysis of the stromal specific dataset is that, despite the wealth of data showing that the stroma can greatly impact cancer progression, few datasets exist that specifically compare stromal gene expression with patient outcome. The stromal dataset examined here contained approximately 50 patients, which has limited statistical power to examine the association between gene expression and patient survival. Analysis of data from TCGA with a larger cohort showed similar trends to the analyses of the breast cancer stroma samples but were not as strongly prognostic, likely because TCGA samples are primarily comprised of tumor epithelia with limited amounts of associated stroma. Therefore, it is possible that hits in this screen influence patient survival but were not captured in this study. Future analyses using more and/or larger datasets may identify additional genes associated with patient outcomes related to the hits identified by this screen. Furthermore, overall survival is complex, and it is perhaps not surprising that more of the hits from a phenotypic screen of kinase inhibitors (in which many inhibitors have broad spectrum effects) did not directly translate to gene expression and correlation with patient survival. Further validation studies will be needed to identify the specific genes, proteins, and pathways that regulate matrix organization and eventually patient outcomes.

Several clinical trials are already in place for several of the compounds identified by this screen, supporting the potential of this approach to identify translatable targets (107–109). Two MET inhibitors, crizotinib and cabozantinib, are FDA approved for NSCLC and renal cell carcinoma, respectively, and a wide variety of other MET inhibitors are in clinical trials (110). The data presented here highlight the need to investigate the impact of these inhibitors on the tumor microenvironment and matrix organization. In addition, while specific kinases have been described in association with these inhibitors, small molecules can have off-target effects, and further studies are needed to conclusively prove the role of specific kinases and pathways in matrix alignment. Further studies of the effects of these inhibitors on normal fibroblast matrix production will also be critical, as normal fibroblasts can have an antitumorigenic effect, in part through production and maintenance of a tumor suppressive ECM (111).

Low stromal PTEN expression has previously been identified as a contributing factor to matrix alignment both *in vitro* and *in vivo* (61). *Pten^−^^/^^−^* fibroblasts were used as a model system to produce highly aligned matrices for kinase pharmacologic inhibitor library screening to identify modulators of matrix alignment. A variety of signaling pathways were suggested on the basis of hits from the screen, including several PI3K/AKT pathway inhibitors, which may reflect the *Pten* knockout model used to induce matrix alignment as PTEN normally acts as a negative regulator of the AKT pathway. A genetically engineered *Pten* null mouse model is already established and well characterized (56, 57, 61), enabling follow-up *in vivo* tumor studies to assess how hits from the screen impact matrix alignment *in vivo*, as well as disease progression (i.e., tumor growth, invasion, metastasis) and other components of the tumor microenvironment (i.e., suppressive effects of normal fibroblasts, angiogenesis, immune response). It will also be important to determine whether the inhibitors and pathways identified here indicate broadly applicable mechanisms of matrix alignment or are specific to the *Pten^−^^/^^−^* model system. On the basis of the characterization studies presented here, the NIH/3T3 system stimulated with TGFβ1 is a promising alternative system that may be more broadly applicable. In addition, while two murine cell lines were used in this work, it will be important to validate these studies with human fibroblasts and primary human tumor CAFs, cell types which have previously been shown to be suitable for the production of CDMs and investigation of matrix alignment (19, 112, 113).

In addition to the pharmacologic inhibitor screening conducted in this study, the HTS matrix alignment platform can be easily adapted to other types of screening. Genetic screening (e.g., siRNA, short hairpin RNA, CRISPR-CAS9) is a powerful tool for identifying genes and pathways that are important for a given biological phenomenon. To adapt the HTS matrix alignment platform for genetic screening, the fibroblasts could be genetically modified in a 384-well plate prior to CDM production, and then the HTS alignment assay and analysis conducted. Future iterations of this platform could also be expanded to include high-content imaging, such as quantification of fibroblast activation markers like fibroblast activation protein (refs. 35, 114). In addition, while the HTS platform detailed here focused on ECM fiber organization and alignment, other ECM characteristics could be easily quantified within this framework, such as the overall amount of ECM (through fluorescence intensity or fiber density) as well as ECM fiber characteristics such as width and length. Increases in collagen fiber density, width, and length have been found to correlate with poor overall survival in patients with gastric cancer (115). Furthermore, collagen fiber length is an independent prognostic factor in head and neck and colorectal cancers and correlates with poor clinical outcomes in esophageal cancer (70).

The organization of ECM fibers in a tissue is a fundamental property critical for tissue and cell function. Changes in this organization, such as the fiber alignment observed in many cancers, can disrupt homeostasis and contribute to disease progression. Matrix alignment around tumors correlates with poor patient outcomes in many cancers, including breast cancer (8), PDAC (11), gastric cancer (115), and head and neck, colorectal, and esophageal cancers (70), indicating the importance of this phenomenon and the potentially broad impact of therapeutic agents capable of normalizing alignment. However, previous attempts to target ECM alignment in cancer have shown little clinical benefits. Thus, new methods are needed to identify additional modifiers of matrix alignment. Here, a novel screening platform was developed to enable high-content and HT analyses of ECM fiber alignment, which will facilitate unbiased high-content screening campaigns and accelerate discovery in this area. Using this platform, several novel regulators of matrix organization were identified that were correlated with poor breast cancer patient outcomes. In particular, c-MET and NTRK1 were identified as regulators of matrix alignment and may serve as novel targets within the cancer stroma. Further efforts to screen different classes of proteins beyond kinases will be important to reveal novel signaling pathways that regulate matrix alignment.

## Supplementary Material

Supplementary Data SD1 Figure S1, S2, S3, Table S1 and S2

## References

[1] Amatangelo MD , Bassi DE, Klein-Szanto AJP, Cukierman E. Stroma-derived three-dimensional matrices are necessary and sufficient to promote desmoplastic differentiation of normal fibroblasts. Am J Pathol 2005;167:475–88. 16049333 10.1016/S0002-9440(10)62991-4 PMC1603576

[2] Provenzano PP , Eliceiri KW, Campbell JM, Inman DR, White JG, Keely PJ. Collagen reorganization at the tumor-stromal interface facilitates local invasion. BMC Med 2006;4:38. 17190588 10.1186/1741-7015-4-38 PMC1781458

[3] Condeelis J , Segall JE. Intravital imaging of cell movement in tumours. Nat Rev Cancer 2003;3:921–30. 14737122 10.1038/nrc1231

[4] Riching KM , Cox BL, Salick MR, Pehlke C, Riching AS, Ponik SM, . 3D collagen alignment limits protrusions to enhance breast cancer cell persistence. Biophys J 2014;107:2546–58. 25468334 10.1016/j.bpj.2014.10.035 PMC4255204

[5] Han W , Chen S, Yuan W, Fan Q, Tian J, Wang X, . Oriented collagen fibers direct tumor cell intravasation. Proc Natl Acad Sci U S A 2016;113:11208–13. 27663743 10.1073/pnas.1610347113 PMC5056065

[6] Grossman M , Ben-Chetrit N, Zhuravlev A, Afik R, Bassat E, Solomonov I, . Tumor cell invasion can be blocked by modulators of collagen fibril alignment that control assembly of the extracellular matrix. Cancer Res 2016;76:4249–58. 27221706 10.1158/0008-5472.CAN-15-2813

[7] Sun X , Wu B, Chiang H-C, Deng H, Zhang X, Xiong W, . Tumour DDR1 promotes collagen fibre alignment to instigate immune exclusion. Nature 2021;599:673–8. 34732895 10.1038/s41586-021-04057-2 PMC8839149

[8] Conklin MW , Eickhoff JC, Riching KM, Pehlke CA, Eliceiri KW, Provenzano PP, . Aligned collagen is a prognostic signature for survival in human breast carcinoma. Am J Pathol 2011;178:1221–32. 21356373 10.1016/j.ajpath.2010.11.076 PMC3070581

[9] Esbona K , Yi Y, Saha S, Yu M, Van Doorn RR, Conklin MW, . The presence of cyclooxygenase 2, tumor-associated macrophages, and collagen alignment as prognostic markers for invasive breast carcinoma patients. Am J Pathol 2018;188:559–73. 29429545 10.1016/j.ajpath.2017.10.025 PMC5963475

[10] Bodelon C , Mullooly M, Pfeiffer RM, Fan S, Abubakar M, Lenz P, . Mammary collagen architecture and its association with mammographic density and lesion severity among women undergoing image-guided breast biopsy. Breast Cancer Res 2021;23:105. 34753492 10.1186/s13058-021-01482-z PMC8579610

[11] Drifka CR , Loeffler AG, Mathewson K, Keikhosravi A, Eickhoff JC, Liu Y, . Highly aligned stromal collagen is a negative prognostic factor following pancreatic ductal adenocarcinoma resection. Oncotarget 2016;7:76197–213. 27776346 10.18632/oncotarget.12772 PMC5342807

[12] Nadiarnykh O , LaComb RB, Brewer MA, Campagnola PJ. Alterations of the extracellular matrix in ovarian cancer studied by Second Harmonic Generation imaging microscopy. BMC Cancer 2010;10:94. 20222963 10.1186/1471-2407-10-94 PMC2841668

[13] Goetz JG , Minguet S, Navarro-Lérida I, Lazcano JJ, Samaniego R, Calvo E, . Biomechanical remodeling of the microenvironment by stromal caveolin-1 favors tumor invasion and metastasis. Cell 2011;146:148–63. 21729786 10.1016/j.cell.2011.05.040 PMC3244213

[14] Birk JW , Tadros M, Moezardalan K, Nadyarnykh O, Forouhar F, Anderson J, . Second harmonic generation imaging distinguishes both high-grade dysplasia and cancer from normal colonic mucosa. Dig Dis Sci 2014;59:1529–34. 24744180 10.1007/s10620-014-3121-7

[15] Kaushik S , Pickup MW, Weaver VM. From transformation to metastasis: deconstructing the extracellular matrix in breast cancer. Cancer Metastasis Rev 2016;35:655–67. 27914000 10.1007/s10555-016-9650-0 PMC5215979

[16] Gritsenko PG , Ilina O, Friedl P. Interstitial guidance of cancer invasion. J Pathol 2012;226:185–99. 22006671 10.1002/path.3031

[17] Cukierman E , Pankov R, Stevens DR, Yamada KM. Taking cell-matrix adhesions to the third dimension. Science 2001;294:1708–12. 11721053 10.1126/science.1064829

[18] Lee H-O , Mullins SR, Franco-Barraza J, Valianou M, Cukierman E, Cheng JD. FAP-overexpressing fibroblasts produce an extracellular matrix that enhances invasive velocity and directionality of pancreatic cancer cells. BMC Cancer 2011;11:245. 21668992 10.1186/1471-2407-11-245 PMC3141768

[19] Castelló-Cros R , Khan DR, Simons J, Valianou M, Cukierman E. Staged stromal extracellular 3D matrices differentially regulate breast cancer cell responses through PI3K and beta1-integrins. BMC Cancer 2009;9:94. 19323811 10.1186/1471-2407-9-94 PMC2669806

[20] Erdogan B , Ao M, White LM, Means AL, Brewer BM, Yang L, . Cancer-associated fibroblasts promote directional cancer cell migration by aligning fibronectin. J Cell Biol 2017;216:3799–816. 29021221 10.1083/jcb.201704053 PMC5674895

[21] Carey SP , Goldblatt ZE, Martin KE, Romero B, Williams RM, Reinhart-King CA. Local extracellular matrix alignment directs cellular protrusion dynamics and migration through Rac1 and FAK. Integr Biol 2016;8:821–35. 10.1039/c6ib00030d PMC4980151 27384462

[22] Sundararaghavan HG , Saunders RL, Hammer DA, Burdick JA. Fiber alignment directs cell motility over chemotactic gradients. Biotechnol Bioeng 2013;110:1249–54. 23172355 10.1002/bit.24788

[23] Quiros RM , Valianou M, Kwon Y, Brown KM, Godwin AK, Cukierman E. Ovarian normal and tumor-associated fibroblasts retain *in vivo* stromal characteristics in a 3-D matrix-dependent manner. Gynecol Oncol 2008;110:99–109. 18448156 10.1016/j.ygyno.2008.03.006 PMC2612536

[24] Alexander JI , Vendramini-Costa DB, Francescone R, Luong T, Franco-Barraza J, Shah N, . Palladin isoforms 3 and 4 regulate cancer-associated fibroblast pro-tumor functions in pancreatic ductal adenocarcinoma. Sci Rep 2021;11:3802. 33589694 10.1038/s41598-021-82937-3 PMC7884442

[25] Özdemir BC , Pentcheva-Hoang T, Carstens JL, Zheng X, Wu C-C, Simpson TR, . Depletion of carcinoma-associated fibroblasts and fibrosis induces immunosuppression and accelerates pancreas cancer with reduced survival. Cancer Cell 2014;25:719–34. 24856586 10.1016/j.ccr.2014.04.005 PMC4180632

[26] Rhim AD , Oberstein PE, Thomas DH, Mirek ET, Palermo CF, Sastra SA, . Stromal elements act to restrain, rather than support, pancreatic ductal adenocarcinoma. Cancer Cell 2014;25:735–47. 24856585 10.1016/j.ccr.2014.04.021 PMC4096698

[27] Kim EJ , Sahai V, Abel EV, Griffith KA, Greenson JK, Takebe N, . Pilot clinical trial of hedgehog pathway inhibitor GDC-0449 (vismodegib) in combination with gemcitabine in patients with metastatic pancreatic adenocarcinoma. Clin Cancer Res 2014;20:5937–45. 25278454 10.1158/1078-0432.CCR-14-1269 PMC4254161

[28] Bijlsma MF , van LHWM. The conflicting roles of tumor stroma in pancreatic cancer and their contribution to the failure of clinical trials: a systematic review and critical appraisal. Cancer Metastasis Rev 2015;34:97–114. 25566685 10.1007/s10555-014-9541-1

[29] Lang M . Infinity Pharma halts pancreatic cancer trial. Boston Bus J; 2012. Available from: https://www.bizjournals.com/boston/blog/mass-high-tech/2012/01/infinity-pharma-halts-pancreatic-cancer-trial.html.

[30] Stylianopoulos T , Munn LL, Jain RK. Reengineering the physical microenvironment of tumors to improve drug delivery and efficacy: from mathematical modeling to bench to bedside. Trends Cancer 2018;4:292–319. 29606314 10.1016/j.trecan.2018.02.005 PMC5930008

[31] Belli C , Trapani D, Viale G, D'Amico P, Duso BA, Vigna PD, . Targeting the microenvironment in solid tumors. Cancer Treat Rev 2018;65:22–32. 29502037 10.1016/j.ctrv.2018.02.004

[32] Peyrol S , Raccurt M, Gerard F, Gleyzal C, Grimaud JA, Sommer P. Lysyl oxidase gene expression in the stromal reaction to *in situ* and invasive ductal breast carcinoma. Am J Pathol 1997;150:497–507. 9033266 PMC1858268

[33] Levental KR , Yu H, Kass L, Lakins JN, Egeblad M, Erler JT, . Matrix crosslinking forces tumor progression by enhancing integrin signaling. Cell 2009;139:891–906. 19931152 10.1016/j.cell.2009.10.027 PMC2788004

[34] Cox TR , Gartland A, Erler JT. Lysyl oxidase, a targetable secreted molecule involved in cancer metastasis. Cancer Res 2016;76:188–92. 26732355 10.1158/0008-5472.CAN-15-2306

[35] Franco-Barraza J , Francescone R, Luong T, Shah N, Madhani R, Cukierman G, . Matrix-regulated integrin αvβ5 maintains α5β1-dependent desmoplastic traits prognostic of neoplastic recurrence. Elife 2017;6:e20600. 28139197 10.7554/eLife.20600 PMC5283834

[36] Gullberg D , Tingström A, Thuresson AC, Olsson L, Terracio L, Borg TK, . Beta 1 integrin-mediated collagen gel contraction is stimulated by PDGF. Exp Cell Res 1990;186:264–72. 2298242 10.1016/0014-4827(90)90305-t

[37] Schiro JA , Chan BM, Roswit WT, Kassner PD, Pentland AP, Hemler ME, . Integrin alpha 2 beta 1 (VLA-2) mediates reorganization and contraction of collagen matrices by human cells. Cell 1991;67:403–10. 1913826 10.1016/0092-8674(91)90191-z

[38] Grundström G , Mosher DF, Sakai T, Rubin K. Integrin alphavbeta3 mediates platelet-derived growth factor-BB-stimulated collagen gel contraction in cells expressing signaling deficient integrin alpha2beta1. Exp Cell Res 2003;291:463–73. 14644167 10.1016/j.yexcr.2003.07.010

[39] Shieh AC , Rozansky HA, Hinz B, Swartz MA. Tumor cell invasion is promoted by interstitial flow-induced matrix priming by stromal fibroblasts. Cancer Res 2011;71:790–800. 21245098 10.1158/0008-5472.CAN-10-1513

[40] Provenzano PP , Inman DR, Eliceiri KW, Trier SM, Keely PJ. Contact guidance mediated three-dimensional cell migration is regulated by rho/ROCK-dependent matrix reorganization. Biophys J 2008;95:5374–84. 18775961 10.1529/biophysj.108.133116 PMC2586586

[41] Kim A , Lakshman N, Petroll WM. Quantitative assessment of local collagen matrix remodeling in 3-D culture: the role of Rho kinase. Exp Cell Res 2006;312:3683–92. 16978606 10.1016/j.yexcr.2006.08.009 PMC2075357

[42] Lemmon CA , Chen CS, Romer LH. Cell traction forces direct fibronectin matrix assembly. Biophys J 2009;96:729–38. 19167317 10.1016/j.bpj.2008.10.009 PMC2716473

[43] Zimmer AS , Steeg PS. Meaningful prevention of breast cancer metastasis: candidate therapeutics, preclinical validation, and clinical trial concerns. J Mol Med 2015;93:13–29. 25412774 10.1007/s00109-014-1226-2 PMC6545582

[44] Raab-Westphal S , Marshall JF, Goodman SL. Integrins as therapeutic targets: successes and cancers. Cancers 2017;9:110. 28832494 10.3390/cancers9090110 PMC5615325

[45] Wei L , Surma M, Shi S, Lambert-Cheatham N, Shi J. Novel insights into the roles of rho kinase in cancer. Arch Immunol Ther Exp 2016;64:259–78. 10.1007/s00005-015-0382-6 PMC4930737 26725045

[46] Feng Y , LoGrasso PV, Defert O, Li R. Rho kinase (ROCK) inhibitors and their therapeutic potential. J Med Chem 2016;59:2269–300. 26486225 10.1021/acs.jmedchem.5b00683

[47] Chin VT , Nagrial AM, Chou A, Biankin AV, Gill AJ, Timpson P, . Rho-associated kinase signalling and the cancer microenvironment: novel biological implications and therapeutic opportunities. Expert Rev Mol Med 2015;17:e17. 26507949 10.1017/erm.2015.17 PMC4836205

[48] Chin VT , Vennin C, Timpson P, Pajic M. Effective modulation of stromal signaling through ROCK inhibition: is it all in the timing? Mol Cell Oncol 2017;4:e1333973. 29057302 10.1080/23723556.2017.1333973 PMC5644476

[49] Jain RK . Normalizing tumor microenvironment to treat cancer: bench to bedside to biomarkers. J Clin Oncol 2013;31:2205–18. 23669226 10.1200/JCO.2012.46.3653 PMC3731977

[50] Gupta GP , Massagué J. Cancer metastasis: building a framework. Cell 2006;127:679–95. 17110329 10.1016/j.cell.2006.11.001

[51] Norton L , Massagué J. Is cancer a disease of self-seeding? Nat Med 2006;12:875–8. 16892025 10.1038/nm0806-875

[52] Ng CP , Swartz MA. Mechanisms of interstitial flow-induced remodeling of fibroblast-collagen cultures. Ann Biomed Eng 2006;34:446–54. 16482410 10.1007/s10439-005-9067-3

[53] Avendano A , Chang JJ, Cortes-Medina MG, Seibel AJ, Admasu BR, Boutelle CM, . Integrated biophysical characterization of fibrillar collagen-based hydrogels. ACS Biomater Sci Eng 2020;6:1408–17. 32292818 10.1021/acsbiomaterials.9b01873 PMC7156078

[54] de Clerck YA , Jones PA. The effect of ascorbic acid on the nature and production of collagen and elastin by rat smooth-muscle cells. Biochem J 1980;186:217–25. 7370010 10.1042/bj1860217 PMC1161522

[55] Serebriiskii I , Castelló-Cros R, Lamb A, Golemis EA, Cukierman E. Fibroblast-derived 3D matrix differentially regulates the growth and drug-responsiveness of human cancer cells. Matrix Biol 2008;27:573–85. 18411046 10.1016/j.matbio.2008.02.008 PMC2603546

[56] Trimboli AJ , Cantemir-Stone CZ, Li F, Wallace JA, Merchant A, Creasap N, . Pten in stromal fibroblasts suppresses mammary epithelial tumours. Nature 2009;461:1084–91. 19847259 10.1038/nature08486 PMC2767301

[57] Bronisz A , Godlewski J, Wallace JA, Merchant AS, Nowicki MO, Mathsyaraja H, . Reprogramming of the tumour microenvironment by stromal PTEN-regulated miR-320. Nat Cell Biol 2012;14:159–67. 10.1038/ncb2396 PMC3271169 22179046

[58] Sizemore G , Balakrishnan S, Hammer A, Thies K, Trimboli A, Wallace J, . Stromal PTEN inhibits the expansion of mammary epithelial stem cells through Jagged-1. Oncogene 2017;36:2297–308. 27797378 10.1038/onc.2016.383 PMC5398932

[59] Franco-Barraza J , Beacham D, Amatangelo MD, Cukierman E. Preparation of extracellular matrices produced by cultured and primary fibroblasts. Curr Protoc Cell Biol 2016;71:10.9.1–34. 10.1002/cpcb.2 PMC5058441 27245425

[60] Pankov R , Momchilova A. Fluorescent labeling techniques for investigation of fibronectin fibrillogenesis (labeling fibronectin fibrillogenesis). Methods Mol Biol 2009;522:261–74. 19247612 10.1007/978-1-59745-413-1_18

[61] Jones CE , Hammer AM, Cho Y, Sizemore GM, Cukierman E, Yee LD, . Stromal PTEN regulates extracellular matrix organization in the mammary gland. Neoplasia 2019;21:132–45. 30550871 10.1016/j.neo.2018.10.010 PMC6293034

[62] Sage D , Prodanov D, Tinevez J-Y, Schindelin J. MIJ: Making interoperability between imagej and matlab possible; 2022. Available from: https://www.mathworks.com/matlabcentral/fileexchange/47545-mij-running-imagej-and-fiji-within-matlab), MATLAB Central File Exchange Retrieved September 21, 2022.

[63] Zhang XD . Illustration of SSMD, z Score, SSMD*, z* score, and t statistic for hit selection in RNAi high-throughput screens. J Biomol Screen 2011;16:775–85. 21515799 10.1177/1087057111405851

[64] Boersma BJ , Reimers M, Yi M, Ludwig JA, Luke BT, Stephens RM, . A stromal gene signature associated with inflammatory breast cancer. Int J Cancer 2008;122:1324–32. 17999412 10.1002/ijc.23237

[65] Cerami E , Gao J, Dogrusoz U, Gross BE, Sumer SO, Aksoy BA, . The cBio cancer genomics portal: an open platform for exploring multidimensional cancer genomics data. Cancer Discov 2012;2:401–4. 22588877 10.1158/2159-8290.CD-12-0095 PMC3956037

[66] Gao J , Aksoy BA, Dogrusoz U, Dresdner G, Gross B, Sumer SO, . Integrative analysis of complex cancer genomics and clinical profiles using the cBioPortal. Sci Signal 2013;6:pl1. 23550210 10.1126/scisignal.2004088 PMC4160307

[67] Gupta V , Bassi DE, Simons JD, Devarajan K, Al-Saleem T, Uzzo RG, . Elevated expression of stromal palladin predicts poor clinical outcome in renal cell carcinoma. PLoS One 2011;6:e21494. 21738681 10.1371/journal.pone.0021494 PMC3125241

[68] Kalluri R , Zeisberg M. Fibroblasts in cancer. Nat Rev Cancer 2006;6:392–401. 16572188 10.1038/nrc1877

[69] Tauriello DVF , Sancho E, Batlle E. Overcoming TGFβ-mediated immune evasion in cancer. Nat Rev Cancer 2022;22:25–44. 34671117 10.1038/s41568-021-00413-6

[70] Hanley CJ , Noble F, Ward M, Bullock M, Drifka C, Mellone M, . A subset of myofibroblastic cancer-associated fibroblasts regulate collagen fiber elongation, which is prognostic in multiple cancers. Oncotarget 2016;7:6159–74. 26716418 10.18632/oncotarget.6740 PMC4868747

[71] Bray MA , Carpenter A, Imaging Platform, Broad Institute of MIT and Harvard. Advanced Assay Development Guidelines for Image-Based High Content Screening and Analysis. 2017 Jul 8. In: Markossian S, Grossman A, Brimacombe K, ., editors. Assay Guidance Manual [Internet]. Bethesda, MD: Eli Lilly & Company and the National Center for Advancing Translational Sciences; 2004–. Available from: https://www-ncbi-nlm-nih-gov.proxy.lib.ohio-state.edu/books/NBK126174/23469374

[72] Godeau AL , Delanoë-Ayari H, Riveline D. Generation of fluorescent cell-derived-matrix to study 3D cell migration. Methods Cell Biol 2020;156:185–203. 32222219 10.1016/bs.mcb.2019.11.013

[73] Jones CE , Sharick JT, Colbert SE, Shukla VC, Zent JM, Ostrowski MC, . Pten regulates collagen fibrillogenesis by fibroblasts through SPARC. PLoS One 2021;16:e0245653. 33534863 10.1371/journal.pone.0245653 PMC7857610

[74] Baneyx G , Baugh L, Vogel V. Coexisting conformations of fibronectin in cell culture imaged using fluorescence resonance energy transfer. Proc Natl Acad Sci U S A 2001;98:14464–8. 11717404 10.1073/pnas.251422998 PMC64704

[75] Rezakhaniha R , Agianniotis A, Schrauwen JTC, Griffa A, Sage D, Bouten CVC, . Experimental investigation of collagen waviness and orientation in the arterial adventitia using confocal laser scanning microscopy. Biomech Model Mechanobiol 2012;11:461–73. 21744269 10.1007/s10237-011-0325-z

[76] Riento K , Ridley AJ. Rocks: multifunctional kinases in cell behaviour. Nat Rev Mol Cell Biol 2003;4:446–56. 12778124 10.1038/nrm1128

[77] Skhirtladze C , Distler O, Dees C, Akhmetshina A, Busch N, Venalis P, . Src kinases in systemic sclerosis: central roles in fibroblast activation and in skin fibrosis. Arthritis Rheum 2008;58:1475–84. 18438865 10.1002/art.23436

[78] Hopkins AL , Groom CR. The druggable genome. Nat Rev Drug Discov 2002;1:727–30. 12209152 10.1038/nrd892

[79] Thies KA , Lefler JE, Leone G, Ostrowski MC. PTEN in the stroma. Cold Spring Harb Perspect Med 2019;9:a036111. 31427286 10.1101/cshperspect.a036111 PMC6771362

[80] Kurose K , Gilley K, Matsumoto S, Watson PH, Zhou X-P, Eng C. Frequent somatic mutations in PTEN and TP53 are mutually exclusive in the stroma of breast carcinomas. Nat Genet 2002;32:355–7. 12379854 10.1038/ng1013

[81] Daikoku T , Jackson L, Besnard V, Whitsett J, Ellenson LH, Dey SK. Cell-specific conditional deletion of Pten in the uterus results in differential phenotypes. Gynecol Oncol 2011;122:424–9. 21570712 10.1016/j.ygyno.2011.04.022 PMC3139002

[82] Ashida S , Orloff MS, Bebek G, Zhang L, Zheng P, Peehl DM, . Integrated analysis reveals critical genomic regions in prostate tumor microenvironment associated with clinicopathologic phenotypes. Clin Cancer Res 2012;18:1578–87. 22275508 10.1158/1078-0432.CCR-11-2535

[83] Sizemore GM , Balakrishnan S, Thies KA, Hammer AM, Sizemore ST, Trimboli AJ, . Stromal PTEN determines mammary epithelial response to radiotherapy. Nat Commun 2018;9:2783. 30018330 10.1038/s41467-018-05266-6 PMC6050339

[84] Pitarresi JR , Liu X, Avendano A, Thies KA, Sizemore GM, Hammer AM, . Disruption of stromal hedgehog signaling initiates RNF5-mediated proteasomal degradation of PTEN and accelerates pancreatic tumor growth. Life Sci Alliance 2018;1:e201800190. 30456390 10.26508/lsa.201800190 PMC6238420

[85] Riedemann J , Macaulay VM. IGF1R signalling and its inhibition. Endocr Relat Cancer 2006;13:S33–43. 17259557 10.1677/erc.1.01280

[86] Hung CF , Rohani MG, Lee S, Chen P, Schnapp LM. Role of IGF-1 pathway in lung fibroblast activation. Respir Res 2013;14:102. 24103846 10.1186/1465-9921-14-102 PMC3840605

[87] Chetty A , Cao G-J, Nielsen HC. Insulin-like growth factor-I signaling mechanisms, type I collagen and alpha smooth muscle actin in human fetal lung fibroblasts. Pediatr Res 2006;60:389–94. 16940243 10.1203/01.pdr.0000238257.15502.f4

[88] Swaney JS , Patel HH, Yokoyama U, Head BP, Roth DM, Insel PA. Focal adhesions in (Myo)fibroblasts scaffold adenylyl cyclase with phosphorylated caveolin. J Biol Chem 2006;281:17173–9. 16618703 10.1074/jbc.M513097200

[89] Li P , Wang D, Lucas J, Oparil S, Xing D, Cao X, . Atrial natriuretic peptide inhibits transforming growth factor β–induced smad signaling and myofibroblast transformation in mouse cardiac fibroblasts. Circ Res 2008;102:185–92. 17991884 10.1161/CIRCRESAHA.107.157677

[90] Miller CL , Cai Y, Oikawa M, Thomas T, Dostmann WR, Zaccolo M, . Cyclic nucleotide phosphodiesterase 1A: a key regulator of cardiac fibroblast activation and extracellular matrix remodeling in the heart. Basic Res Cardiol 2011;106:1023–39. 22012077 10.1007/s00395-011-0228-2 PMC4114346

[91] Matei A-E , Beyer C, Györfi A-H, Soare A, Chen C-W, Dees C, . Protein kinases G are essential downstream mediators of the antifibrotic effects of sGC stimulators. Ann Rheum Dis 2018;77:459. 29311148 10.1136/annrheumdis-2017-212489

[92] Wójcik-Pszczoła K , Chłoń-Rzepa G, Jankowska A, Ślusarczyk M, Ferdek PE, Kusiak AA, . A novel, pan-PDE inhibitor exerts anti-fibrotic effects in human lung fibroblasts via inhibition of TGF-β signaling and activation of cAMP/PKA signaling. Int J Mol Sci 2020;21:4008. 32503342 10.3390/ijms21114008 PMC7312375

[93] Delaunay M , Osman H, Kaiser S, Diviani D. The role of cyclic AMP signaling in cardiac fibrosis. Cells 2019;9:69. 31888098 10.3390/cells9010069 PMC7016856

[94] Qin L , Zang M, Xu Y, Zhao R, Wang Y, Mi Y, . Chlorogenic acid alleviates hyperglycemia-induced cardiac fibrosis through activation of the NO/cGMP/PKG pathway in cardiac fibroblasts. Mol Nutr Food Res 2021;65:2000810. 10.1002/mnfr.202000810 33200558

[95] Finak G , Bertos N, Pepin F, Sadekova S, Souleimanova M, Zhao H, . Stromal gene expression predicts clinical outcome in breast cancer. Nat Med 2008;14:518–27. 18438415 10.1038/nm1764

[96] Faress JA , Nethery DE, Kern EFO, Eisenberg R, Jacono FJ, Allen CL, . Bleomycin-induced pulmonary fibrosis is attenuated by a monoclonal antibody targeting HER2. J Appl Physiol 2007;103:2077–83. 17916677 10.1152/japplphysiol.00239.2007

[97] Andrianifahanana M , Wilkes MC, Gupta SK, Rahimi RA, Repellin CE, Edens M, . Profibrotic TGFβ responses require the cooperative action of PDGF and ErbB receptor tyrosine kinases. FASEB J 2013;27:4444–54. 23913859 10.1096/fj.12-224907 PMC3804746

[98] Li H , Shao F, Qian B, Sun Y, Huang Z, Ding Z, . Upregulation of HER2 in tubular epithelial cell drives fibroblast activation and renal fibrosis. Kidney Int 2019;96:674–88. 31327474 10.1016/j.kint.2019.04.012

[99] Humeres C , Shinde AV, Hanna A, Alex L, Hernández SC, Li R, . Smad7 effects on TGF-β and ErbB2 restrain myofibroblast activation and protect from postinfarction heart failure. J Clin Invest 2022;132:e146926. 34905511 10.1172/JCI146926 PMC8803336

[100] Palazzo E , Marconi A, Truzzi F, Dallaglio K, Petrachi T, Humbert P, . Role of neurotrophins on dermal fibroblast survival and differentiation. J Cell Physiol 2012;227:1017–25. 21503896 10.1002/jcp.22811

[101] Hang P-Z , Ge F-Q, Li P-F, Liu J, Zhu H, Zhao J. The regulatory role of the BDNF/TrkB pathway in organ and tissue fibrosis. Histol Histopathol 2021;36:1133–43. 34327702 10.14670/HH-18-368

[102] Edakuni G , Sasatomi E, Satoh T, Tokunaga O, Miyazaki K. Expression of the hepatocyte growth factor/c-Met pathway is increased at the cancer front in breast carcinoma. Pathol Int 2001;51:172–8. 11328532 10.1046/j.1440-1827.2001.01182.x

[103] Birchmeier C , Birchmeier W, Gherardi E, Woude GFV. Met, metastasis, motility and more. Nat Rev Mol Cell Biol 2003;4:915–25. 14685170 10.1038/nrm1261

[104] Peruzzi B . Targeting the c-Met signaling pathway in cancer. Clin Cancer Res 2006;12:3657–60. 16778093 10.1158/1078-0432.CCR-06-0818

[105] Mochly-Rosen D , Das K, Grimes KV. Protein kinase C, an elusive therapeutic target? Nat Rev Drug Discov 2012;11:937–57. 23197040 10.1038/nrd3871 PMC3760692

[106] Bahar C , Stuhlmann D, Stelnbrenner H, Alili L, Holtkötter O, Sies H, . Enhancement of tumor invasion depends on transdifferentiation of skin fibroblasts mediated by reactive oxygen species. J Cell Sci 2006;119:2727–38. 16757516 10.1242/jcs.03011

[107] Dorff TB , Quinn DI, Pinski JK, Goldkorn A, Sadeghi S, Tsao-Wei D, . Randomized phase II trial of abiraterone alone or with dasatinib in men with metastatic castration-resistant prostate cancer (mCRPC). Clin Genitourin Cancer 2019;17:241–7. 31227432 10.1016/j.clgc.2019.02.010 PMC7446934

[108] Morris PG , Rota S, Cadoo K, Zamora S, Patil S, D'Andrea G, . Phase II study of paclitaxel and dasatinib in metastatic breast cancer. Clin Breast Cancer 2018;18:387–94. 29680193 10.1016/j.clbc.2018.03.010 PMC6682312

[109] Mitri Z , Nanda R, Blackwell K, Costelloe CM, Hood I, Wei C, . TBCRC-010: phase I/II study of dasatinib in combination with zoledronic acid for the treatment of breast cancer bone metastasis. Clin Cancer Res 2016;22:5706–12. 27166393 10.1158/1078-0432.CCR-15-2845

[110] Hughes VS , Siemann DW. Have clinical trials properly assessed c-Met inhibitors? Trends Cancer 2018;4:94–7. 29458966 10.1016/j.trecan.2017.11.009 PMC5824436

[111] Alkasalias T , Moyano-Galceran L, Arsenian-Henriksson M, Lehti K. Fibroblasts in the tumor microenvironment: shield or spear? Int J Mol Sci 2018;19:1532. 29883428 10.3390/ijms19051532 PMC5983719

[112] Soucy PA , Romer LH. Endothelial cell adhesion, signaling, and morphogenesis in fibroblast-derived matrix. Matrix Biol 2009;28:273–83. 19375504 10.1016/j.matbio.2009.04.005

[113] Scherzer MT , Waigel S, Donninger H, Arumugam V, Zacharias W, Clark G, . Fibroblast-derived extracellular matrices: an alternative cell culture system that increases metastatic cellular properties. PLoS One 2015;10:e0138065. 26371754 10.1371/journal.pone.0138065 PMC4570771

[114] Sandberg TP , Stuart M, Oosting J, Tollenaar R, Sier CFM, Mesker WE. Increased expression of cancer-associated fibroblast markers at the invasive front and its association with tumor-stroma ratio in colorectal cancer. BMC Cancer 2019;19:284. 30922247 10.1186/s12885-019-5462-2 PMC6440123

[115] Zhou Z-H , Ji C-D, Xiao H-L, Zhao H-B, Cui Y-H, Bian X-W. Reorganized collagen in the tumor microenvironment of gastric cancer and its association with prognosis. J Cancer 2017;8:1466–76. 28638462 10.7150/jca.18466 PMC5479253

