# Supplementary material for: A Miniaturized Screening Platform to Identify Novel Regulators of Extracellular Matrix Alignment
Source: Cancer Res Commun. 2022 Nov 22;2(11):1471–86. doi: 10.1158/2767-9764.CRC-22-0157 (PMC9757767; doi:10.1158/2767-9764.CRC-22-0157)
Supplement: Supplementary Data SD1 — Figure S1, S2, S3, Table S1 and S2 [file crc-22-0157-s01.docx]

**Supplemental Information**

**
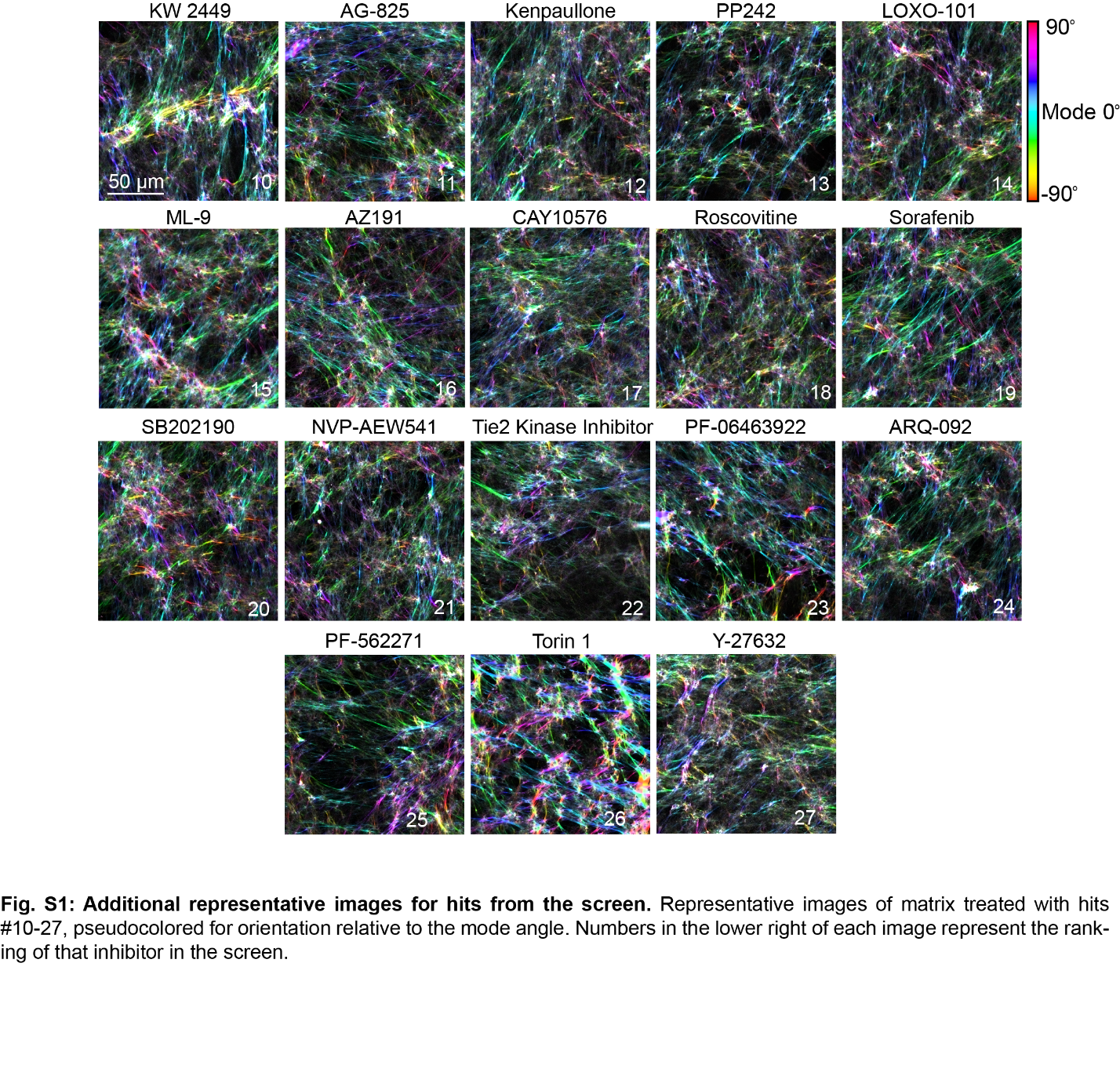

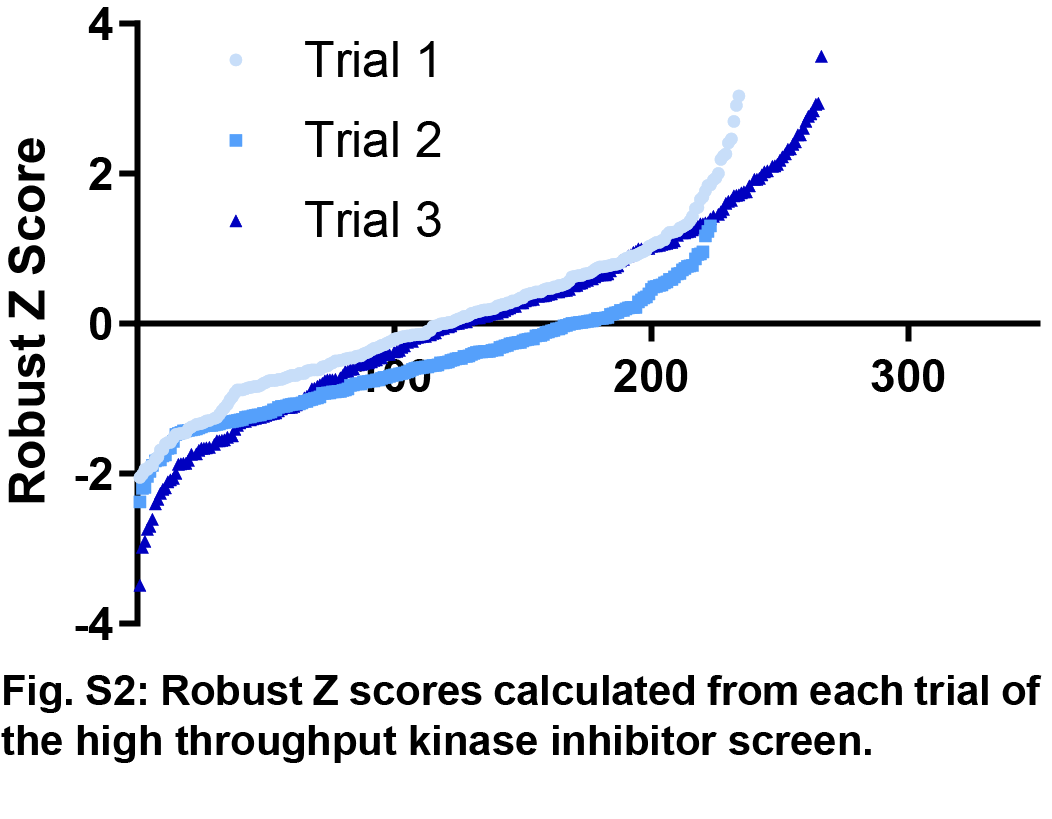
**

**Fig. S2: Robust Z scores calculated from each trial of the high throughput kinase inhibitor screen.**

**
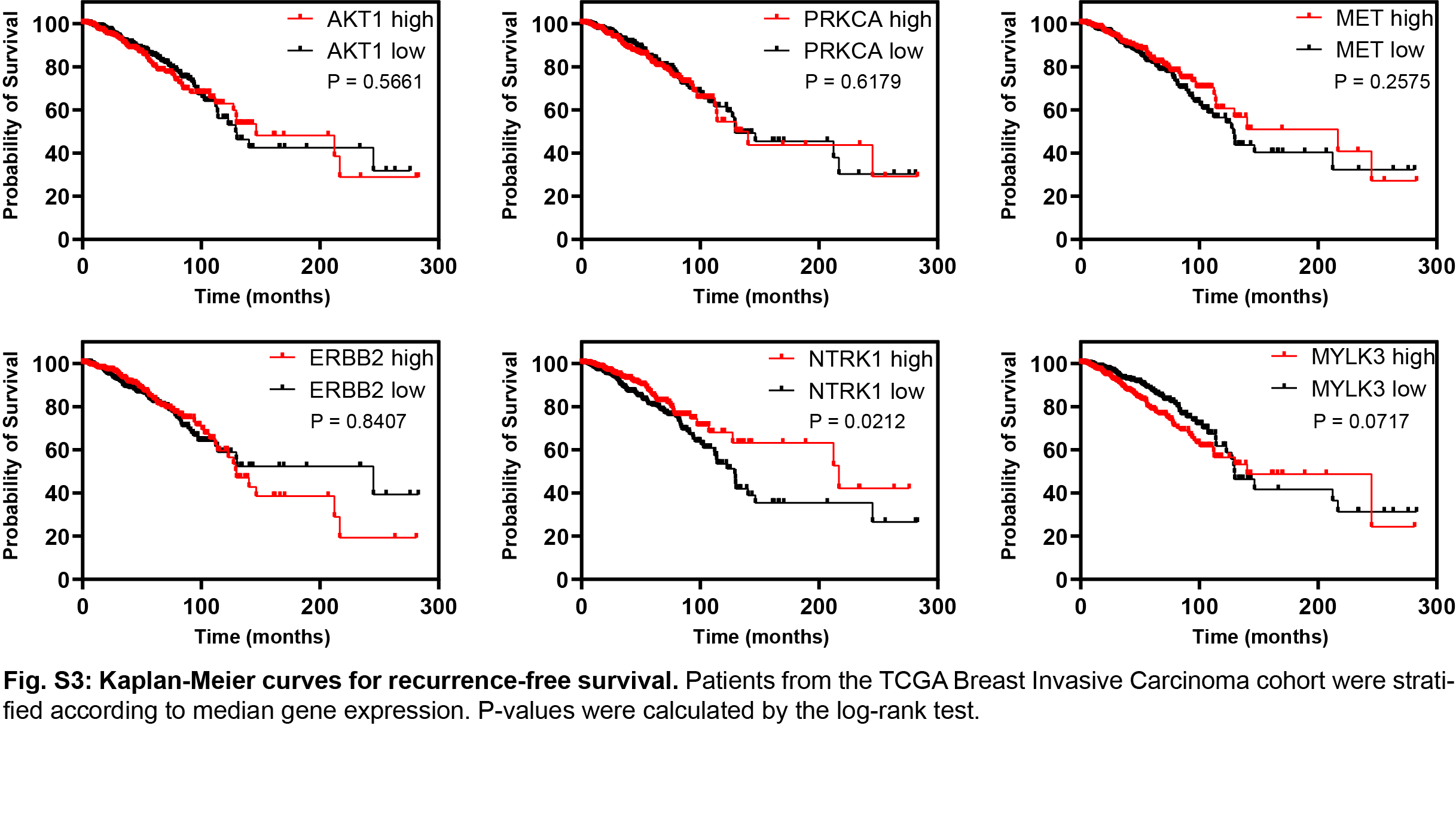
**

**Supplemental Table 1. Screening data from all inhibitors.** Robust Z-score mean and S.D. only include wells with sufficient matrix and cell viability. Nuclei counts, a metric of cell viability, are the average of all 3 screen replicates, regardless of whether sufficient matrix and cell viability was found.

| **Drug and Dose** | **Target** | **Robust Z-score** | **S.D.** | **# Valid Screens** | **% of Control Nuclei** |
| --- | --- | --- | --- | --- | --- |
| Afuresertib(hydrochloride) 10µM | PKB/Akt | -3.488 | n/a | 1 | 47.4 |
| BX-912 10µM | PDK1 | -2.906 | n/a | 1 | 37.1 |
| LCK Inhibitor 10µM | LcK | -2.108 | n/a | 1 | 54.6 |
| H-9(hydrochloride) 10µM | PKG | -1.969 | 0.737 | 3 | 83.8 |
| URMC-099 1µM | LRRK2, MLKs | -1.788 | 0.421 | 2 | 66.2 |
| ARQ-092 10µM | PKB/Akt | -1.758 | 0.683 | 3 | 133.5 |
| AZD 1208 1µM | PIMs | -1.682 | 0.700 | 3 | 78.5 |
| Gö 6983 10µM | PKC | -1.679 | 0.836 | 3 | 79.7 |
| AP26113 10µM | ALK | -1.674 | 0.269 | 3 | 80.8 |
| Tie2 Kinase Inhibitor 10µM | Tie2 | -1.658 | n/a | 1 | 61.2 |
| R406 10µM | Syk | -1.649 | n/a | 1 | 50.4 |
| SC-1 10µM | ERK1 | -1.633 | 0.113 | 2 | 68.9 |
| (R)-Crizotinib 1µM | c-Met, ALK | -1.540 | 0.271 | 2 | 87.4 |
| Bisindolylmaleimide I 10µM | PKC | -1.517 | 0.231 | 3 | 68.5 |
| KW 2449 1µM | FLT3, ABL | -1.449 | 0.145 | 3 | 62.4 |
| AG-825 10µM | ErbB2 | -1.427 | 0.139 | 2 | 104.0 |
| BIBF 1120 10µM | Multi-kinase | -1.420 | n/a | 1 | 52.6 |
| Kenpaullone 10µM | GSK3B, CDKs | -1.391 | 1.110 | 3 | 100.9 |
| PP242 1µM | mTOR | -1.387 | 1.017 | 2 | 56.1 |
| LOXO-101 1µM | Trk family | -1.352 | 0.449 | 3 | 102.0 |
| ML-9 10µM | Multi-kinase | -1.301 | 0.406 | 3 | 103.9 |
| AZ191 1µM | DYRK1b | -1.299 | 0.118 | 3 | 99.0 |
| Bosutinib 10µM | c-Src, Abl | -1.294 | n/a | 1 | 75.4 |
| CAY10576 1µM | IKKε | -1.214 | 0.382 | 3 | 80.2 |
| (R)-Roscovitine 1µM | CDKs | -1.193 | 0.718 | 3 | 110.7 |
| JNJ-10198409 10µM | PDGFR | -1.192 | n/a | 1 | 43.4 |
| Sorafenib 1µM | Raf-1 | -1.175 | 0.868 | 3 | 121.2 |
| SB 202190 10µM | p38 MAPK | -1.163 | 0.403 | 2 | 77.9 |
| NVP-AEW541(hydrochloride) 1µM | IGF-1R | -1.146 | 0.664 | 3 | 88.5 |
| GSK2334470 10µM | PDK1 | -1.081 | n/a | 1 | 45.2 |
| Tie2 Kinase Inhibitor 1µM | Tie2 | -1.078 | 0.374 | 3 | 86.8 |
| PF-06463922 10µM | ALK | -1.072 | 0.807 | 2 | 67.7 |
| ARQ-092 1µM | PKB/Akt | -1.026 | 0.321 | 3 | 80.9 |
| PF-562271(besylate) 1µM | FAK | -1.009 | 0.245 | 3 | 70.0 |
| Lavendustin C 10µM | EGFR | -0.989 | n/a | 1 | 65.5 |
| Torin 1 1µM | mTOR | -0.980 | 0.051 | 2 | 61.8 |
| Y-27632(hydrochloride) 1µM | ROCK-1 | -0.976 | 0.923 | 2 | 83.8 |
| AG-494 1µM | EGFR | -0.909 | 0.703 | 3 | 92.3 |
| BMS-777607 10µM | Met family, Axl family | -0.876 | 0.898 | 3 | 57.7 |
| PI3-Kinase α Inhibitor 2 10µM | PI3Ka | -0.872 | 0.620 | 3 | 54.5 |
| AP26113 1µM | ALK | -0.870 | 0.994 | 3 | 82.2 |
| PD 169316 10µM | p38 MAPK | -0.868 | 0.423 | 3 | 102.9 |
| H-9(hydrochloride) 1µM | PKG | -0.788 | 1.054 | 3 | 84.4 |
| Indirubin-3'-monoxime 10µM | GSK3b | -0.784 | 0.321 | 2 | 107.6 |
| LY364947 10µM | ALK5 | -0.779 | 1.005 | 3 | 92.4 |
| PI-103 1µM | DNA-PK, PI3K, mTOR | -0.773 | 0.946 | 3 | 92.7 |
| Wortmannin 1µM | PI3K | -0.772 | 0.785 | 3 | 97.4 |
| Sorafenib 10µM | Raf-1 | -0.746 | 0.608 | 2 | 75.8 |
| NU 6102 10µM | Cdk1/2 | -0.744 | n/a | 1 | 78.6 |
| AZ 3146 10µM | Mps1 | -0.739 | 0.950 | 3 | 102.9 |
| SB-216763 1µM | GSK3 | -0.737 | 0.249 | 3 | 106.1 |
| Sunitinib(malate) 10µM | FLK1, PDGFRb | -0.733 | n/a | 1 | 54.2 |
| Afuresertib(hydrochloride) 1µM | PKB/Akt | -0.710 | 0.854 | 3 | 91.1 |
| Gö 6983 1µM | PKC | -0.708 | 0.780 | 3 | 98.2 |
| Bosutinib 1µM | c-Src, Abl | -0.704 | 1.241 | 2 | 114.2 |
| SB-431542(hydrate) 10µM | ALK5 | -0.701 | 0.874 | 3 | 83.6 |
| TAS 120 10µM | FGFR family | -0.699 | 0.337 | 3 | 65.1 |
| JNJ-10198409 1µM | PDGFR | -0.691 | 0.280 | 2 | 53.5 |
| (R)-Roscovitine 10µM | CDKs | -0.683 | 0.733 | 3 | 103.4 |
| GSK2334470 1µM | PDK1 | -0.670 | 0.378 | 3 | 106.2 |
| Bisindolylmaleimide I 1µM | PKC | -0.665 | 0.487 | 3 | 103.5 |
| PD 173074 1µM | FGFR1 | -0.660 | 0.499 | 3 | 95.7 |
| CRT0066101(hydrochloride) 1µM | PKD | -0.651 | 0.809 | 3 | 99.8 |
| HA-1077(hydrochloride) 1µM | ROCK II | -0.646 | 0.093 | 3 | 91.9 |
| U-0126 1µM | MEK 1/2 | -0.638 | 0.398 | 3 | 100.4 |
| FRAX597 1µM | PAK1/2/3 | -0.630 | 0.893 | 3 | 96.8 |
| GW 5074 10µM | Raf-1 | -0.630 | 0.649 | 3 | 113.3 |
| BMS-5 10µM | LIMK 1/2 | -0.626 | 0.969 | 3 | 87.0 |
| NVP-TAE226 1µM | FAK, PYK2b | -0.617 | 0.518 | 3 | 79.4 |
| CAY10626 1µM | PI3Ka, mTOR | -0.612 | n/a | 1 | 58.9 |
| KN-93 10µM | CaMKII | -0.606 | 0.330 | 3 | 102.2 |
| Ruxolitinib 1µM | JAK 1/2 | -0.592 | 0.471 | 3 | 104.3 |
| U-0126 10µM | MEK 1/2 | -0.592 | 0.458 | 3 | 89.4 |
| Torin 1 10µM | mTOR | -0.563 | 0.431 | 2 | 57.4 |
| Sunitinib(malate) 1µM | FLK1, PDGFRb | -0.552 | 1.223 | 3 | 74.8 |
| CAY10622 10µM | ROCK 1/2 | -0.544 | 0.138 | 3 | 104.0 |
| Bisindolylmaleimide V 10µM | S6K | -0.532 | 0.101 | 2 | 91.9 |
| AG-183 10µM | EGFR | -0.530 | 0.705 | 3 | 89.7 |
| Bisindolylmaleimide IX(mesylate) 1µM | PKC, GSK2 | -0.530 | 0.448 | 3 | 109.7 |
| BMS-777607 1µM | Met family, Axl family | -0.514 | 1.069 | 3 | 92.0 |
| AS-605240 1µM | PI3Ky | -0.500 | 1.368 | 2 | 93.7 |
| Tilfrinib 1µM | BRK/PTK6 | -0.496 | 0.460 | 3 | 97.6 |
| PHA-665752 10µM | c-Met | -0.491 | 1.050 | 3 | 63.4 |
| LFM-A13 10µM | BTK | -0.490 | 0.684 | 3 | 96.8 |
| AZD 0156 1µM | ATM | -0.482 | 0.493 | 3 | 71.8 |
| Leelamine 10µM | PDK | -0.475 | 0.877 | 3 | 76.2 |
| PF-06463922 1µM | ALK | -0.474 | 0.895 | 3 | 97.8 |
| TGX-221 10µM | PI3K p110b | -0.473 | 0.047 | 3 | 95.6 |
| YM-201636 1µM | PIKfyve | -0.448 | 1.030 | 2 | 80.8 |
| Chelerythrine(chloride) 1µM | PCK | -0.438 | 1.625 | 3 | 88.8 |
| KN-93 1µM | CaMKII | -0.438 | 0.449 | 3 | 107.1 |
| SMI-4a 10µM | PIMs | -0.429 | 1.073 | 3 | 89.0 |
| SU6656 10µM | Yes | -0.427 | 0.301 | 2 | 46.2 |
| GSK1059615 1µM | PI3Ka | -0.422 | 1.096 | 3 | 90.4 |
| SP 600125 1µM | JNKs | -0.402 | 0.501 | 3 | 106.4 |
| SB-431542(hydrate) 1µM | ALK5 | -0.388 | 1.529 | 3 | 97.4 |
| WZ4003 10µM | NUAK1/2 | -0.379 | 0.747 | 3 | 89.6 |
| H-89(hydrochloride) 1µM | Multi-kinase | -0.378 | 0.378 | 3 | 104.0 |
| Lavendustin C 1µM | EGFR | -0.377 | 0.778 | 3 | 110.5 |
| BI-D1870 10µM | RSKs | -0.373 | n/a | 1 | 53.8 |
| GW 5074 1µM | Raf-1 | -0.373 | 1.063 | 3 | 104.2 |
| Mps1-IN-1 10µM | Mps1 | -0.369 | 0.751 | 3 | 78.1 |
| Olomoucine 1µM | CDKs | -0.357 | 0.865 | 2 | 88.6 |
| BMS-5 1µM | LIMK 1/2 | -0.357 | 0.010 | 2 | 91.0 |
| NVP-TAE226 10µM | FAK, PYK2b | -0.352 | n/a | 1 | 30.0 |
| H-8(hydrochloride) 10µM | PKA, PKG | -0.341 | 0.287 | 3 | 81.5 |
| SC-514 1µM | IKK2 | -0.331 | 0.661 | 3 | 109.6 |
| VE-822 1µM | ATR | -0.330 | 0.461 | 3 | 72.7 |
| AG-879 1µM | BMX | -0.328 | 0.906 | 3 | 84.5 |
| Indirubin-3'-monoxime 1µM | GSK3b | -0.322 | 0.667 | 3 | 103.3 |
| Canertinib(hydrochloride) 1µM | EGFR | -0.320 | 0.344 | 3 | 106.4 |
| BIBF 1120 1µM | Multi-kinase | -0.320 | n/a | 1 | 65.5 |
| SU 6668 1µM | Aurora B | -0.320 | 0.418 | 3 | 106.7 |
| SB 203580 1µM | p38 MAPK | -0.304 | 0.453 | 3 | 117.5 |
| AG-494 10µM | EGFR | -0.296 | 0.688 | 3 | 97.7 |
| TGX-221 1µM | PI3K p110b | -0.296 | 1.142 | 3 | 98.0 |
| AG-490 10µM | JAK2 | -0.292 | 0.679 | 3 | 91.7 |
| Phthalazinone pyrazole 1µM | Aurora a | -0.289 | 0.366 | 3 | 104.2 |
| (S)-H-1152(hydrochloride) 10µM | ROCK | -0.274 | 0.919 | 3 | 87.8 |
| SC-514 10µM | IKK2 | -0.267 | 0.685 | 3 | 104.3 |
| LCK Inhibitor 1µM | LcK | -0.265 | 0.235 | 3 | 94.7 |
| LY2606368 1µM | Chk1 | -0.254 | 0.687 | 3 | 53.3 |
| PI-103 10µM | DNA-PK, PI3K, mTOR | -0.253 | n/a | 1 | 42.4 |
| BX-912 1µM | PDK1 | -0.240 | 1.147 | 3 | 98.1 |
| TWS119 1µM | GSK3b | -0.235 | 0.471 | 3 | 102.4 |
| Apatinib 1µM | VEGFR2 | -0.218 | 0.879 | 3 | 93.5 |
| Nilotinib 1µM | Bcr-Abl | -0.217 | 0.793 | 3 | 105.8 |
| CHIR99021 1µM | GSK3 | -0.215 | 1.013 | 3 | 103.2 |
| Syk Inhibitor II 1µM | Syk | -0.208 | 0.814 | 3 | 101.4 |
| Apatinib 10µM | VEGFR2 | -0.208 | 0.479 | 3 | 82.2 |
| CAY10505 10µM | CK2 | -0.206 | 0.513 | 3 | 97.6 |
| 17β-hydroxy Wortmannin 10µM | PI3K | -0.205 | 0.195 | 3 | 79.2 |
| KN-62 10µM | CaMKII | -0.201 | 0.669 | 3 | 83.2 |
| ML-9 1µM | Multi-kinase | -0.186 | 0.158 | 3 | 108.6 |
| Erbstatin analog 1µM | EGFR | -0.180 | 0.425 | 3 | 101.2 |
| PD 0325901 1µM | MEK | -0.174 | 1.863 | 2 | 61.4 |
| GNE-7915 10µM | LRRK2 | -0.168 | 1.389 | 3 | 90.3 |
| MLCK Inhibitor Peptide 18 10µM | MLCK | -0.168 | 0.524 | 3 | 108.1 |
| LOXO-101 10µM | Trk family | -0.162 | 0.189 | 3 | 92.5 |
| Mps1-IN-1 1µM | Mps1 | -0.159 | 0.622 | 3 | 93.0 |
| Erlotinib 1µM | EGFR | -0.149 | 0.228 | 2 | 94.5 |
| SU6656 1µM | Yes | -0.119 | 0.193 | 3 | 91.2 |
| XMD16-5 1µM | TNK2 | -0.088 | 0.415 | 3 | 74.8 |
| CAY10505 1µM | CK2 | -0.088 | 0.781 | 3 | 109.1 |
| LOXO-195 1µM | Trk family | -0.084 | 0.828 | 3 | 95.4 |
| Sphingosine Kinase Inhibitor 2 10µM | SPHK1 | -0.079 | 0.572 | 3 | 74.0 |
| VX-702 1µM | p38 MAPK | -0.069 | 0.825 | 3 | 106.2 |
| PP2 1µM | LYN, Fyn, HCK | -0.068 | 1.063 | 3 | 99.3 |
| MLCK Inhibitor Peptide 18 1µM | MLCK | -0.061 | 1.552 | 3 | 93.5 |
| PD 184161 1µM | MEK 1/2 | -0.045 | 0.605 | 3 | 101.8 |
| Erbstatin analog 10µM | EGFR | -0.041 | 0.003 | 2 | 77.6 |
| Sphingosine Kinase Inhibitor 2 1µM | SPHK1 | -0.029 | 0.430 | 3 | 100.1 |
| AG-825 1µM | ErbB2 | -0.021 | 1.020 | 3 | 100.7 |
| CAY10621 10µM | PSPHK1 | 0.005 | 0.438 | 2 | 95.7 |
| CHIR99021 10µM | GSK3 | 0.009 | 0.182 | 2 | 73.6 |
| 3-Methyladenine 10µM | PI3K | 0.015 | 0.852 | 3 | 104.5 |
| Nilotinib 10µM | Bcr-Abl | 0.023 | 0.621 | 3 | 98.3 |
| RPI-1 10µM | RET | 0.035 | 0.760 | 3 | 93.2 |
| 17β-hydroxy Wortmannin 1µM | PI3K | 0.053 | 1.207 | 3 | 91.1 |
| PD 184161 10µM | MEK 1/2 | 0.062 | n/a | 1 | 40.8 |
| 5-Iodotubercidin 1µM | CK1, PKC, ERK2 | 0.071 | 1.676 | 3 | 86.4 |
| Kenpaullone 1µM | GSK3B, CDKs | 0.075 | 0.653 | 3 | 109.1 |
| AZ 3146 1µM | Mps1 | 0.076 | 1.427 | 3 | 81.3 |
| CAY10578 10µM | CK2 | 0.080 | n/a | 1 | 77.9 |
| Wortmannin 10µM | PI3K | 0.082 | 0.122 | 3 | 86.3 |
| AG-1478 10µM | EGFR | 0.083 | 0.485 | 3 | 81.7 |
| 3-Methyladenine 1µM | PI3K | 0.085 | 0.888 | 3 | 101.2 |
| AS-703026 10µM | MEK 1/2 | 0.093 | 1.208 | 2 | 47.3 |
| ZM 447439 1µM | Aurora B | 0.093 | 0.676 | 3 | 98.4 |
| SMI-4a 1µM | PIMs | 0.098 | 0.528 | 2 | 97.3 |
| AS-703026 1µM | MEK 1/2 | 0.104 | 0.929 | 3 | 79.0 |
| AS-605240 10µM | PI3Ky | 0.114 | 0.137 | 3 | 117.0 |
| CAY10621 1µM | PSPHK1 | 0.117 | 0.063 | 2 | 103.4 |
| SB-216763 10µM | GSK3 | 0.129 | 1.906 | 2 | 92.8 |
| BGJ398 10µM | FGFR family | 0.137 | 0.945 | 3 | 66.6 |
| AS-041164 1µM | PI3K y | 0.186 | 0.498 | 3 | 79.1 |
| Emodin 1µM | CK2 | 0.191 | 0.606 | 2 | 99.2 |
| AG-183 1µM | EGFR | 0.199 | 1.541 | 3 | 102.0 |
| Chelerythrine(chloride) 10µM | PCK | 0.207 | 1.510 | 2 | 72.0 |
| PP2 10µM | LYN, Fyn, HCK | 0.218 | 0.246 | 2 | 78.4 |
| Doramapimod 10µM | p38 MAPK | 0.220 | 0.526 | 3 | 134.5 |
| AS-041164 10µM | PI3K y | 0.231 | 0.618 | 2 | 76.2 |
| CAY10578 1µM | CK2 | 0.237 | 0.616 | 3 | 107.8 |
| PD 0325901 10µM | MEK | 0.246 | 0.415 | 2 | 40.7 |
| Leelamine 1µM | PDK | 0.263 | 1.218 | 3 | 99.2 |
| CGP 57380 1µM | MNK1 | 0.288 | 1.145 | 3 | 112.5 |
| GNE-7915 1µM | LRRK2 | 0.301 | 0.845 | 3 | 98.1 |
| CAY10574 10µM | Cdk9 | 0.317 | 0.370 | 3 | 92.5 |
| CAY10561 1µM | ERK2 | 0.328 | 1.685 | 3 | 83.0 |
| WHI-P131 10µM | JAK3 | 0.333 | 0.333 | 3 | 86.4 |
| RG-13022 1µM | EGFR | 0.337 | 1.142 | 3 | 100.3 |
| PD 169316 1µM | p38 MAPK | 0.358 | 0.908 | 3 | 108.6 |
| UNC569 1µM | Axl family | 0.361 | 0.564 | 3 | 96.6 |
| AG-370 10µM | PDGFR | 0.362 | 0.675 | 2 | 85.4 |
| Doramapimod 1µM | p38 MAPK | 0.379 | 0.242 | 3 | 103.7 |
| AG-1296 1µM | PDGFR | 0.383 | 0.284 | 3 | 98.9 |
| Tilfrinib 10µM | BRK/PTK6 | 0.385 | n/a | 1 | 51.7 |
| Erlotinib 10µM | EGFR | 0.395 | 0.525 | 3 | 75.2 |
| CAY10576 10µM | IKKε | 0.406 | n/a | 1 | 50.6 |
| PHA-665752 1µM | c-Met | 0.437 | 1.384 | 3 | 92.9 |
| GNF-5 10µM | Bcr-Abl | 0.441 | 0.864 | 3 | 92.0 |
| WHI-P131 1µM | JAK3 | 0.442 | 1.158 | 3 | 98.2 |
| GNF-5 1µM | Bcr-Abl | 0.450 | 1.445 | 3 | 106.9 |
| TG003 1µM | CLK1, CLK4 | 0.454 | 1.307 | 3 | 84.3 |
| KRN 633 10µM | VEGFR family | 0.455 | 0.142 | 3 | 77.8 |
| SU 6668 10µM | Aurora B | 0.459 | 0.406 | 3 | 81.9 |
| Olomoucine 10µM | CDKs | 0.460 | 0.428 | 3 | 100.4 |
| PI3-Kinase α Inhibitor 2 1µM | PI3Ka | 0.476 | 0.425 | 3 | 95.2 |
| PD 173074 10µM | FGFR1 | 0.477 | 1.988 | 2 | 102.7 |
| ABT-869 1µM | VEGFR family, PDGFR family | 0.507 | 0.869 | 3 | 89.7 |
| RPI-1 1µM | RET | 0.507 | 1.252 | 2 | 77.4 |
| BIO 1µM | GSK3 | 0.508 | 0.793 | 3 | 106.5 |
| TAS 120 1µM | FGFR family | 0.520 | 1.107 | 3 | 87.7 |
| CAY10622 1µM | ROCK 1/2 | 0.526 | 0.771 | 3 | 106.4 |
| SB 202190 1µM | p38 MAPK | 0.530 | 0.577 | 3 | 113.8 |
| (S)-H-1152(hydrochloride) 1µM | ROCK | 0.531 | 0.377 | 3 | 97.3 |
| WZ4003 1µM | NUAK1/2 | 0.540 | 0.362 | 2 | 90.4 |
| AG-1478 1µM | EGFR | 0.545 | 0.513 | 3 | 98.4 |
| LY364947 1µM | ALK5 | 0.555 | 0.868 | 3 | 102.6 |
| CAY10561 10µM | ERK2 | 0.561 | 0.179 | 3 | 70.7 |
| JNK Inhibitor XVI 1µM | JNKs | 0.572 | 0.578 | 3 | 107.5 |
| Bisindolylmaleimide V 1µM | S6K | 0.606 | 1.029 | 3 | 101.4 |
| NU 6102 1µM | Cdk1/2 | 0.608 | 0.545 | 3 | 108.1 |
| R406 1µM | Syk | 0.618 | 0.623 | 3 | 78.4 |
| H-8(hydrochloride) 1µM | PKA, PKG | 0.626 | 1.051 | 3 | 86.3 |
| INK128 1µM | mTOR | 0.629 | 0.546 | 2 | 64.1 |
| CGP 57380 10µM | MNK1 | 0.653 | 0.478 | 3 | 75.1 |
| AG-370 1µM | PDGFR | 0.680 | 0.303 | 2 | 103.4 |
| ABT-869 10µM | VEGFR family, PDGFR family | 0.686 | 2.050 | 3 | 72.0 |
| Imatinib(mesylate) 1µM | PDGFR, c-Kit | 0.688 | 1.524 | 3 | 96.8 |
| BMS 345541(hydrochloride) 1µM | IKKb | 0.714 | 1.134 | 3 | 81.7 |
| Syk Inhibitor II 10µM | Syk | 0.715 | 0.202 | 2 | 50.0 |
| CAY10574 1µM | Cdk9 | 0.731 | 1.521 | 3 | 112.2 |
| TG003 10µM | CLK1, CLK4 | 0.764 | 0.468 | 3 | 119.3 |
| CAY10571 10µM | p38 MAPK | 0.786 | 1.484 | 3 | 103.3 |
| LOXO-195 10µM | Trk family | 0.805 | 0.554 | 3 | 97.3 |
| BGJ398 1µM | FGFR family | 0.814 | 1.510 | 3 | 76.7 |
| Necrostatin-1 1µM | RIP1 | 0.830 | 1.496 | 3 | 109.9 |
| Necrostatin-1 10µM | RIP1 | 0.845 | 1.054 | 3 | 109.4 |
| Imatinib(mesylate) 10µM | PDGFR, c-Kit | 0.862 | 0.996 | 3 | 90.7 |
| AZD 7762 1µM | Chk 1/2 | 0.867 | n/a | 1 | 51.4 |
| PLX4032 10µM | B-RAF | 0.908 | 0.089 | 3 | 103.1 |
| D 4476 1µM | CK1 | 0.909 | 1.504 | 3 | 105.9 |
| KN-62 1µM | CaMKII | 0.935 | 1.180 | 3 | 94.0 |
| Vatalanib(hydrochloride) 1µM | VEGFR family | 0.951 | 0.479 | 2 | 82.1 |
| VX-702 10µM | p38 MAPK | 0.963 | 0.783 | 3 | 114.1 |
| LFM-A13 1µM | BTK | 0.996 | 1.501 | 3 | 95.6 |
| BI-D1870 1µM | RSKs | 1.011 | 1.372 | 3 | 104.5 |
| PLX4032 1µM | B-RAF | 1.054 | 1.673 | 3 | 102.8 |
| Vatalanib(hydrochloride) 10µM | VEGFR family | 1.060 | 1.261 | 2 | 87.4 |
| AG-490 1µM | JAK2 | 1.077 | 1.062 | 3 | 101.2 |
| SB 203580 10µM | p38 MAPK | 1.095 | 1.425 | 3 | 101.0 |
| SP 600125 10µM | JNKs | 1.107 | 0.762 | 3 | 84.6 |
| Emodin 10µM | CK2 | 1.139 | 1.342 | 3 | 99.6 |
| CAY10571 1µM | p38 MAPK | 1.301 | 0.696 | 3 | 101.5 |
| AG-1296 10µM | PDGFR | 1.368 | 1.437 | 3 | 97.6 |
| RG-13022 10µM | EGFR | 1.372 | 0.973 | 3 | 102.9 |
| KRN 633 1µM | VEGFR family | 1.386 | 0.764 | 3 | 96.8 |
| AG-879 10µM | BMX | 1.473 | 0.764 | 3 | 68.8 |
| D 4476 10µM | CK1 | 1.924 | n/a | 1 | 88.0 |
| (R)-Crizotinib 10µM | c-Met, ALK |  |  | 0 | 39.9 |
| (S)-Glycyl-H-1152(hydrochloride) 1µM | ROCK II |  |  | 0 | 71.7 |
| (S)-Glycyl-H-1152(hydrochloride) 10µM | ROCK II |  |  | 0 | 50.0 |
| 5-Iodotubercidin 10µM | CK1, PKC, ERK2 |  |  | 0 | 43.0 |
| AZ191 10µM | DYRK1b |  |  | 0 | 35.6 |
| AZD 0156 10µM | ATM |  |  | 0 | 22.6 |
| AZD 1208 10µM | PIMs |  |  | 0 | 39.4 |
| AZD 7762 10µM | Chk 1/2 |  |  | 0 | 16.5 |
| BI-6727 1µM | Pilks |  |  | 0 | 46.8 |
| BI-6727 10µM | Pilks |  |  | 0 | 51.9 |
| BIO 10µM | GSK3 |  |  | 0 | 27.5 |
| Bisindolylmaleimide IX(mesylate) 10µM | PKC, GSK2 |  |  | 0 | 47.6 |
| BMS 345541(hydrochloride) 10µM | IKKb |  |  | 0 | 38.7 |
| Canertinib(hydrochloride) 10µM | EGFR |  |  | 0 | 39.9 |
| CAY10626 10µM | PI3Ka, mTOR |  |  | 0 | 25.0 |
| CRT0066101(hydrochloride) 10µM | PKD |  |  | 0 | 52.2 |
| Dasatinib 1µM | Src |  |  | 0 | 65.8 |
| Dasatinib 10µM | Src |  |  | 0 | 39.9 |
| FRAX597 10µM | PAK1/2/3 |  |  | 0 | 50.1 |
| GSK1059615 10µM | PI3Ka |  |  | 0 | 65.7 |
| H-89(hydrochloride) 10µM | Multi-kinase |  |  | 0 | 82.9 |
| HA-1077(hydrochloride) 10µM | ROCK II |  |  | 0 | 49.4 |
| INK128 10µM | mTOR |  |  | 0 | 38.0 |
| JNK Inhibitor XVI 10µM | JNKs |  |  | 0 | 21.5 |
| KW 2449 10µM | FLT3, ABL |  |  | 0 | 60.9 |
| LDN-211904 1µM | EphB3 |  |  | 0 | 67.8 |
| LDN-211904 10µM | EphB3 |  |  | 0 | 43.0 |
| LY2606368 10µM | Chk1 |  |  | 0 | 88.4 |
| NVP-AEW541(hydrochloride) 10µM | IGF-1R |  |  | 0 | 33.0 |
| PF-562271(besylate) 10µM | FAK |  |  | 0 | 69.7 |
| Phthalazinone pyrazole 10µM | Aurora a |  |  | 0 | 23.4 |
| PIK-75(hydrochloride) 1µM | PI3K p110a |  |  | 0 | 22.0 |
| PIK-75(hydrochloride) 10µM | PI3K p110a |  |  | 0 | 40.2 |
| PKC 412 1µM | Multi-kinase |  |  | 0 | 47.9 |
| PKC 412 10µM | Multi-kinase |  |  | 0 | 47.1 |
| PP242 10µM | mTOR |  |  | 0 | 70.2 |
| Ruxolitinib 10µM | JAK 1/2 |  |  | 0 | 52.5 |
| SC-1 1µM | ERK1 |  |  | 0 | 136.5 |
| Staurosporine 1µM | PKC |  |  | 0 | 29.2 |
| Staurosporine 10µM | PKC |  |  | 0 | 27.5 |
| TWS119 10µM | GSK3b |  |  | 0 | 37.2 |
| UNC569 10µM | Axl family |  |  | 0 | 37.4 |
| URMC-099 10µM | LRRK2, MLKs |  |  | 0 | 38.8 |
| VE-822 10µM | ATR |  |  | 0 | 24.0 |
| XMD16-5 10µM | TNK2 |  |  | 0 | 38.5 |
| Y-27632(hydrochloride) 10µM | ROCK-1 |  |  | 0 | 71.2 |
| YM-201636 10µM | PIKfyve |  |  | 0 | 112.9 |
| ZM 447439 10µM | Aurora B |  |  | 0 | 27.0 |

**Supplemental Table 2: Top inhibitors from the matrix alignment screen ranked by robust z-score and association of mRNA expression of related inhibitor protein targets with patient survival.** Gene expression in ductal breast carcinoma stroma was correlated with patient survival at 5-years. Patients were grouped according to their survival status and gene expression was compared by t-test. Fold change represents average gene expression in patients reported as deceased at 5-years relative to those alive at 5-years. Significant changes are highlighted in bold.
